# Advancements in Cardiac Magnetic Resonance Imaging: Innovations, Challenges, and Future Directions

**DOI:** 10.31083/RCM48251

**Published:** 2026-07-22

**Authors:** Jasmine K. Dugal, Roger Woolley, Alexis Liszewski, Rooz Razmi, Sean Nemeh, Jaskirat S. Malhi, Arpinder S. Malhi, Aditi Singh

**Affiliations:** ^1^Department of Internal Medicine, University of Nevada Las Vegas, Las Vegas, NV 89102, USA; ^2^A.T. Still University Kirksville College of Osteopathic Medicine, Kirksville, MO 63501, USA; ^3^Michigan State University College of Osteopathic Medicine, East Lansing, MI 48824, USA; ^4^Department of Radiology, University of Arizona Tucson, Tucson, AZ 85724, USA

**Keywords:** CMR, gadolinium, 4D flow MRI, artificial intelligence, machine learning, hybrid imaging, contrast agents

## Abstract

Cardiac magnetic resonance (CMR) imaging has become one of the most comprehensive noninvasive tools for evaluating cardiac structure, function, and myocardial tissue characteristics. Moreover, the role of CMR imaging in cardiovascular tomography has become increasingly central, providing unparalleled insights across a wide range of cardiac diseases. Over the past decade, several technical advances have expanded the clinical applications of CMR imaging and strengthened the role of this modality in diagnosis and management. Therefore, this review synthesizes these rapid advances and the associated growing influence on contemporary cardiovascular imaging practice. This literature review examines recent advances in CMR imaging, including improved contrast agents, refined mapping techniques, integration of artificial intelligence (AI), and accelerated imaging protocols, and highlights how these innovations are enhancing CMR imaging performance and delivering meaningful benefits in clinical practice. Recent advances in CMR imaging include higher-resolution imaging, novel gadolinium-enhanced and parametric mapping sequences, strain imaging, and four-dimensional (4D) flow magnetic resonance imaging (MRI), enabling more comprehensive assessment of myocardial structure, function, and hemodynamics. Improvements in contrast agents, the development of gadolinium alternatives, and increasing integration of AI have further enhanced the diagnostic accuracy and workflow efficiency. Advances in CMR imaging have strengthened the role of this technique as a highly accurate and increasingly accessible tool for cardiovascular evaluation, despite ongoing challenges related to cost, patient tolerance, and contrast use. Future directions should include enhanced tissue characterization, integration with complementary imaging modalities, and continued technological innovation to expand clinical applications, improve patient outcomes, and reduce reliance on radiation-based techniques.

## 1. Introduction

### 1.1 Global Impact of Cardiovascular Disease

Cardiovascular disease (CVD) is the leading cause of death and disability worldwide, accounting for an estimated 19.2 million deaths and 437 million disability-adjusted life years (DALYs) in 2023 [1]. CVD is responsible for approximately one-third of all global deaths and represents 15.6% of the total global disease burden [1,2]. This burden reflects a range of conditions, including ischemic heart disease, cardiomyopathies, valvular disease, congenital heart disease, and heart failure.

Globally, the prevalence of CVD has risen dramatically, driven by population aging, urbanization, and increasing rates of major cardiovascular risk factors such as hypertension, dyslipidemia, diabetes, and obesity. Prevalent CVD cases more than doubled from 271 million in 1990 to 626 million in 2023, while deaths increased from 12.1 million to 19.2 million [1,2]. Projections indicate that CVD prevalence and mortality will continue to increase through 2050, with some models estimating nearly a 90% increase in CVD prevalence and greater than a 70% increase in deaths if the current trends continue [3,4].

The economic burden of CVD is equally profound and rapidly escalating. In the United States, total and indirect costs of CVD are expected to double from $ 555 billion in 2015 to $ 1.1 trillion by 2035, driven by rising healthcare expenditures and productivity losses [3]. Globally, low- and middle-income countries are expected to have approximately 3.6 trillion dollars in CVD-related economic losses, accounting for nearly half of all noncommunicable disease costs [5]. Without effective prevention and treatment strategies, CVD is projected to cost the global economy up to $ 47 trillion over the next 25 years [5].

These global trends underscore the urgent need for improved strategies in cardiovascular prevention, diagnosis, and management. Advanced imaging modalities like cardiac magnetic resonance imaging (CMR) are therefore becoming increasingly essential for early detection, precise diagnosis, risk stratification, and furthering individualized management aimed at reducing the global burden of CVD.

### 1.2 Overview of CMR

CMR has emerged as a cornerstone in the noninvasive assessment of cardiovascular disease, offering unparalleled visualization of cardiac anatomy, function, perfusion, and tissue characterization without the use of ionizing radiation [6,7]. Since its inception in the 1970s as a tool for metabolic studies, CMR has evolved through major technological milestones. Over the past five decades, developments include advanced imaging sequences, the introduction of gadolinium-based contrast agents, and the adoption of parametric mapping techniques. These developments have allowed CMR to provide a near-comprehensive evaluation of cardiac structure, function, perfusion, and tissue characterization within a single study [6,7,8]. CMR has progressed from a research modality to an indispensable clinical technique, now integrated into major population studies and clinical guidelines worldwide [6,7,8]. Recent innovations, such as artificial intelligence-driven acquisition and analysis, accelerated imaging protocols, and advanced flow and tissue mapping, continue to expand its clinical utility and accessibility [7].

**Fig. 1.**
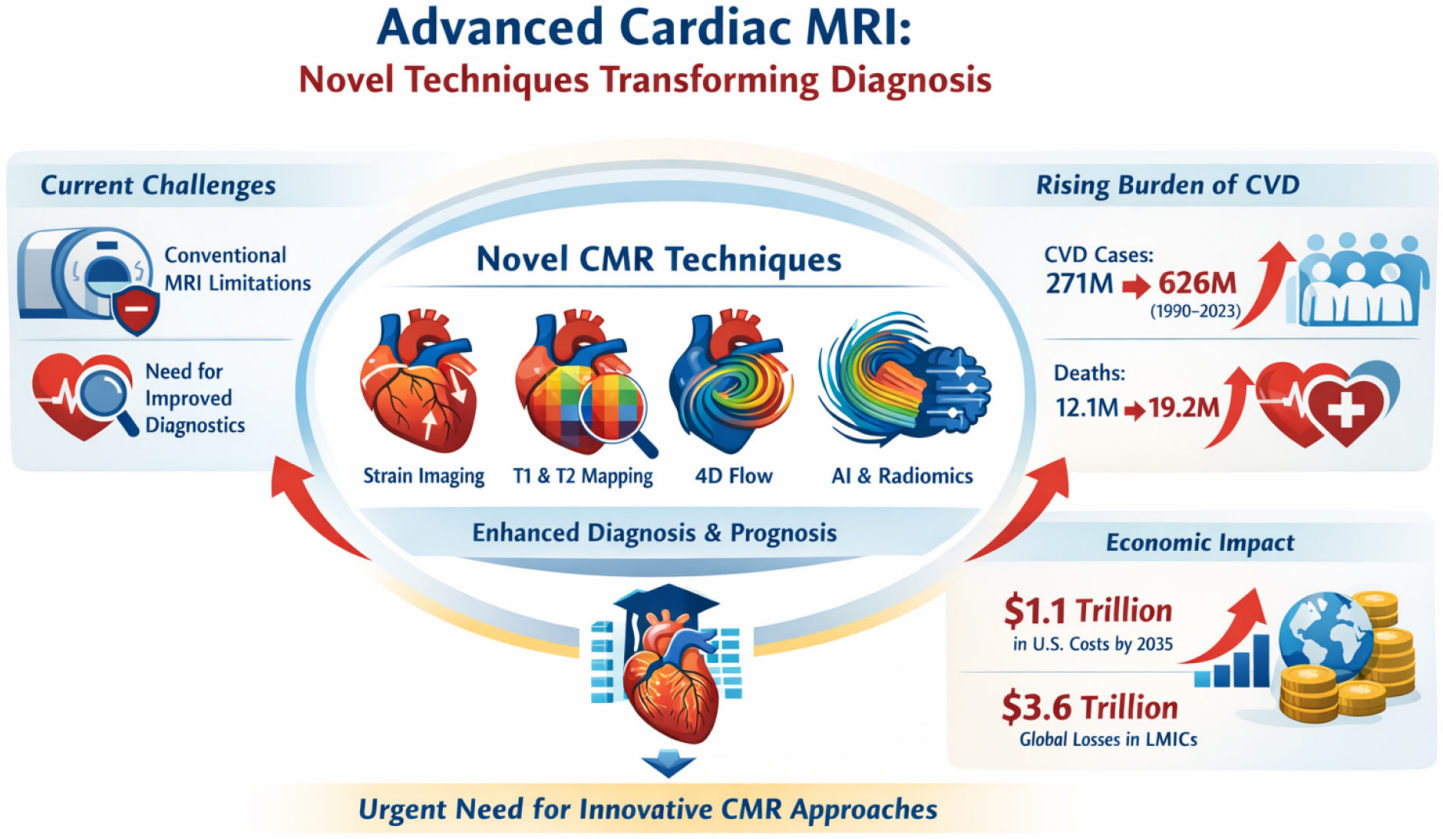
**Advanced cardiac magnetic resonance imaging (MRI): novel techniques transforming diagnosis**. This graphical summary demonstrates the recent advancements in cardiac magnetic resonance (CMR) being discussed in this review, which allows for improved diagnostics in the midst of rising burden of cardiovascular disease (CVD) and its estimated economic impact. 4D, four-dimensional; AI, artificial intelligence.

Compared with echocardiography, computed tomography (CT), and nuclear imaging, CMR provides superior tissue characterization, quantitative flow assessment via four-dimensional (4D) flow, and advanced mapping techniques, all without using ionizing radiation [9]. These unique strengths allow CMR to detect subtle myocardial disease, evaluate complex hemodynamics, and integrate artificial intelligence (AI)-driven analysis for more precise diagnosis and prognostication. This positions CMR to be a uniquely versatile tool in modern cardiovascular disease. Fig. 1 provides a graphical summary of the major limitations with conventional methods in CMR as well as the novel techniques that allow for further advancements in diagnosing and treating cardiovascular diseases.

### 1.3 Purpose of the Review

This review aims to highlight recent technological advancements in CMR, discuss the expanding clinical applications and persistent limitations of CMR, and identify emerging trends and future directions in CMR research, underscoring its pivotal role in contemporary cardiology.

## 2. Advanced CMR Techniques

### 2.1 Imaging Techniques and Sequences

#### 2.1.1 Cine Imaging and High-Resolution Acquisition

Cine CMR captures dynamic cardiac motion to evaluate ventricular volumes, ejection fraction, and wall motion, providing a detailed functional assessment throughout the cardiac cycle [10]. Cardiac imaging requires a rapid capture of moving structures to accurately assess cardiac function, and recent advances in temporal and spatial resolution have significantly improved cine CMR. Modern real-time Fast Low-Angle Shot (FLASH) sequences paired with non-linear inverse reconstruction now achieve temporal resolution of 20–30 milliseconds and spatial resolution of 1.5 millimeters during free breathing, compared with 40–50 milliseconds and 2.0–2.5 millimeters previously [11]. Additionally, golden angle radial and spiral sampling techniques, combined with compressed sensing and low-rank plus sparse reconstruction, enable continuous imaging at 30 to 50 frames per second without breath-holds or electrocardiographic synchronization [11]. These advances allow for accurate assessment of cardiac function in patients who cannot perform the breath hold or those who have arrhythmias. Free-breathing protocols expand the population able to successfully complete CMR exams and improve overall patient tolerance [11,12]. Studies demonstrate that these innovations minimize motion artifacts and improve scan success [11,12]. Limitations include the need for specialized reconstruction algorithms and computational resources, which may not be available in all clinical centers.

#### 2.1.2 Late Gadolinium Enhancement (LGE)

LGE detects myocardial fibrosis and scar by detecting differential gadolinium retention in diseased tissue. It is particularly effective for visualizing focal fibrosis and infarction, though it is less sensitive to diffuse interstitial fibrosis [13]. Optimized LGE sequences improve contrast between normal and diseased myocardium, enabling precise delineation of focal scar and supporting diagnosis, risk stratification, prognosis, and treatment planning in myocardial infarction, cardiomyopathies, and myocarditis. Complementary use of T1 mapping and extracellular volume (ECV) quantification enables assessment of diffuse fibrosis, which provides a more comprehensive evaluation of myocardial tissue health and supports earlier intervention in conditions such as hypertrophic and dilated cardiomyopathy or infiltrative disease [13]. Limitations of LGE include reduced sensitivity for diffuse fibrosis and contraindications in patients with severe renal impairment due to gadolinium exposure [13].

#### 2.1.3 Parametric Mapping (T1 and T2)

CMR parametric mapping techniques, including T1, T2, and ECV assessment, enable quantitative myocardial tissue characterization. Clinically, parametric mapping has broad applications across cardiomyopathies, infiltrative diseases, and ischemic heart disease. Native T1 mapping is critical for evaluating myocardial fibrosis, providing measurements of both native T1 values and ECV, which reflects the expansion of the interstitial space. T2 mapping assesses myocardial edema, offering insight into acute injury and inflammation. Advances in native T1 mapping enable characterization of interstitial fibrosis and edema without contrast, while post-contrast T1 measurements allow calculation of ECV, providing a quantitative measure of diffuse myocardial fibrosis [13]. Improvements in T2 mapping enhance sensitivity to myocardial edema, supporting early detection of inflammatory or ischemic injury [14]. Parametric mapping has proven useful for diagnosing and monitoring infiltrative and fibrotic cardiomyopathies such as amyloidosis, Anderson-Fabry disease and hypertrophic cardiomyopathy [15,16,17]. T2 mapping detects acute myocardial edema in conditions such as myocarditis and ischemic injury [14]. Parametric mapping results in earlier detection of subtle tissue changes that may not be evident on LGE, enabling earlier intervention and more timely management [18,19]. Although there are many benefits to parametric mapping, limitations include reliance on high-quality image acquisition, the need for specialized interpretation expertise, and variability across imaging platforms, highlighting the importance of standardization and local validation [16,20].

#### 2.1.4 Strain Imaging

Strain imaging quantifies regional myocardial deformation, providing a functional assessment of the heart beyond global measures like ejection fraction. Techniques such as feature tracking and tissue tagging allow for a noninvasive measurement of regional myocardial deformation, which enables early detection of subclinical myocardial dysfunction. Combining these advanced sequences provides a comprehensive assessment of both structure and function, enhancing CMR diagnostic confidence across multiple pathologies, especially regarding the functional capacity of the heart [21]. Evidence shows that strain imaging improves sensitivity for detecting early myocardial dysfunction before changes in conventional metrics [21]. Limitations of strain imaging include dependency on imaging quality, long acquisition times, lack of standardization, and measurement variability [22,23,24].

#### 2.1.5 4D Flow MRI

4D flow magnetic resonance imaging (MRI) provides a dynamic, volumetric, and time-resolved assessment of blood flow throughout the cardiac chambers and great vessels while also capturing three-directional velocity-encoded data that enables visualization and quantification of complex flow patterns like vortices, helices, and retrograde jets. It also allows for noninvasive measurement of physiologic parameters, including kinetic energy, wall shear stress, and vorticity, therefore offering insights into cardiovascular hemodynamics without interference artifacts [25]. 4D flow technical developments in compressed sensing and parallel imaging have reduced acquisition times, allowing for more clinical feasibility. Clinically, the integration of 4D flow with machine learning tools has automated segmentation and streamlined flow analysis, increasing 4D flow’s utility in both congenital and acquired heart disease [26]. Additionally, 4D flow improves assessment of valvular disease severity, aortic regurgitation, and intracardiac shunts with high reproducibility [25]. Widespread implementation remains limited by specialized acquisition requirements, computational demands, and the need for standardized protocols [25,26].

#### 2.1.6 Acceleration and Motion Correction Techniques

Motion during scanning can degrade image quality and limit diagnostic accuracy, particularly in pediatric, critically ill, or arrhythmic patients. Techniques such as compressed sensing, parallel imaging, navigator gating, and real-time motion correction enable faster free-breathing scans with reduced artifacts, expanding the feasibility of CMR for patients who cannot cooperate with conventional protocols. While these strategies maintain image fidelity and improve patient tolerability, they require specialized software and expertise that may not be accessible in all centers [11,12,27,28].

### 2.2 Functional MRI

#### 2.2.1 Advances in Functional Assessment, Including Cardiac Motion Analysis

Feature tracking cardiovascular magnetic resonance (FT-CMR) and displacement encoding with stimulated echoes (DENSE) have improved the analysis of strain, displacement, and torsion [7]. FT-CMR derives strain parameters from standard cine images. Without the need for specialized sequences, this increases the clinical accessibility by allowing patients to skip an additional diagnostic measure. Meanwhile, DENSE provides high-resolution displacement data of subtle motion and contractile dysfunction. Together, these methods allow for early detection of cardiomyopathies and are particularly valuable in conditions like hypertrophic cardiomyopathy, where regional strain abnormalities may occur before overt structural changes [29].

#### 2.2.2 Techniques to Assess Myocardial Contractility, Perfusion, and Metabolism

CMR development has improved beyond anatomical and functional imaging to include greater metabolic and perfusion assessment capability. While first-pass perfusion imaging with gadolinium-based contrast continues as the evaluation method of myocardial ischemia and perfusion, particularly during pharmacological stress evaluations, quantitative perfusion mapping goes a step further. Quantitative, pixel-wise perfusion mapping of myocardial blood flow improves the accuracy in the diagnosis of multivessel and microvascular cardiomyopathies [30].

**Fig. 2.**
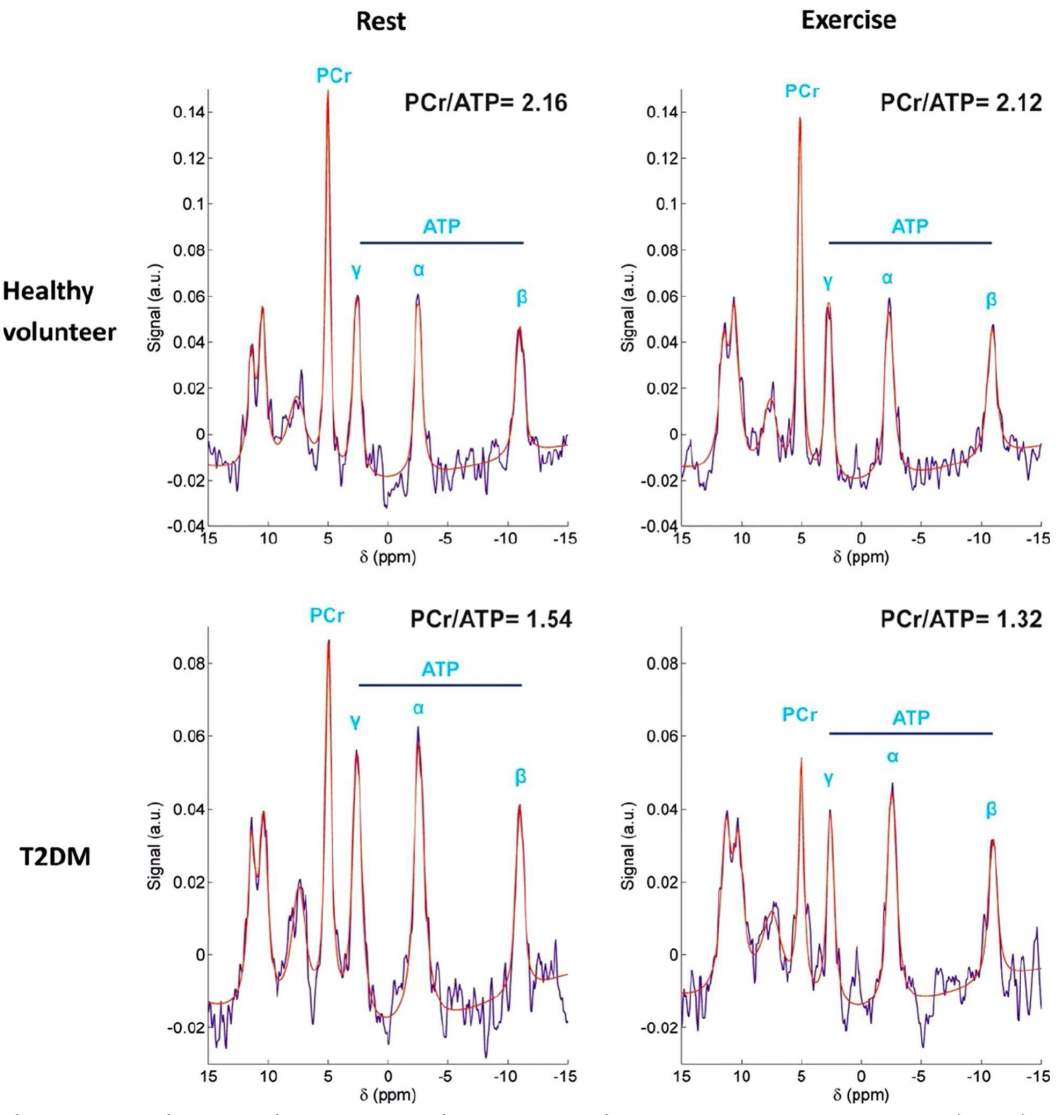
**Myocardial energetic assessment using 31P magnetic resonance spectroscopy**. Rest and exercise myocardial 31P magnetic resonance (31P-MR) spectra in a healthy volunteer and a patient with type 2 diabetes mellitus, illustrating impaired myocardial energetics in the diabetic heart. Reproduced with permission by Springer Nature, Inc [31]. PCr, Phosphocreatine; ATP, Adenosine Triphosphate; T2DM, type 2 diabetes mellitus.

Beyond perfusion, techniques such as blood oxygen level-dependent (BOLD) imaging provide insights into myocardial oxygenation without contrast, identifying ischemic regions based on deoxyhemoglobin content. Simultaneously, magnetic resonance (MR) spectroscopy allows metabolic profiling of myocardial tissue, including high-energy phosphate quantification (e.g., Phosphocreatine (PCr)/Adenosine Triphosphate (ATP) ratio), which may indicate early metabolic derangements in heart failure and diabetic cardiomyopathy (Fig. 2, Ref. [31]) [31].

These modalities enhance the functional profile of cardiac MRI, enabling a multi-parametric approach that evaluates contractility, perfusion, and metabolism in a single session.

### 2.3 Resolution and Speed Improvements

#### 2.3.1 Advancements in Spatial and Temporal Resolution

Further spatial and temporal resolution improvements have enabled more precise imaging of rapidly moving cardiac structures. Traditional temporal resolutions of 40–50 milliseconds can now be reduced below 30 milliseconds through real-time FLASH sequences. This captures finer details of myocardial motion and valvular dynamics. Simultaneously, spatial resolution has improved from 2.0–2.5 mm to approximately 1.5 mm, even during free-breathing acquisitions, minimizing motion artifacts and improving diagnostic reliability [11,32].

Steady-state free precession (SSFP) imaging and FLASH sequences, when paired with reconstruction algorithms, produce high-resolution imaging without breath holding or electrocardiogram (ECG) gating. This makes CMR particularly more useful when imaging pediatric populations with arrhythmias or poor breath-holding ability [28].

#### 2.3.2 Techniques Such as Parallel Imaging and Compressed Sensing to Improve Scan Times and Resolution

Parallel imaging techniques like sensitivity encoding (SENSE) and generalized autocalibrating partially parallel acquisitions (GAPPA) significantly reduce scan time by acquiring multiple lines of k-space data simultaneously. When combined with newer acceleration strategies such as compressed sensing (CS), scan durations are shortened even further while preserving image quality [33].

Real-time cardiac imaging at continuous image acquisition with frame rates of 30–50 frames per second can be achieved through compressed sensing of MR images, which reconstructs high-quality images from undersampled data. This maintains a high temporal resolution without compromising spatial detail [11].

By integrating these technologies, whole-heart imaging and functional sequences can be completed in under 5 minutes. This increases patients’ comfort and likelihood of scan completion while expanding clinical CMR applications.

### 2.4 Contrast Agents and Gadolinium Alternatives

#### 2.4.1 New Developments in Contrast Agents

To increase safety and diagnostic capability, novel contrast agent developments aim to simultaneously increase diagnostic capability and address increased safety concerns with gadolinium-based contrast agents (GBCAs). Currently, GBCAs are classified by the American College of Radiology (ACR) into groups (I–III) based on risk of nephrogenic systemic fibrosis (NSF) (Table 1, Ref. [34]).

**Table 1.** **American College of Radiology (ACR) classification of gadolinium-based contrast agents (GBCAs) according to nephrogenic systemic fibrosis (NSF) risk [34]**.

Group classification	Agents	NSF association
Group I	Gadodiamide (Omniscan), Gadopentetate dimeglumine (Magnevist), Gadoversetamide (OptiMARK)	Greatest number of NSF cases
Group II	Gadobenate dimeglumine (MultiHance), Gadobutrol (Gadavist), Gadoteric acid (Dotarem), Gadoteridol (ProHance)	Few, unconfounded/confirmed NSF cases
Group III	Agents with limited data on NSF risk	Insufficient evidence to classify risk

**Fig. 3.**
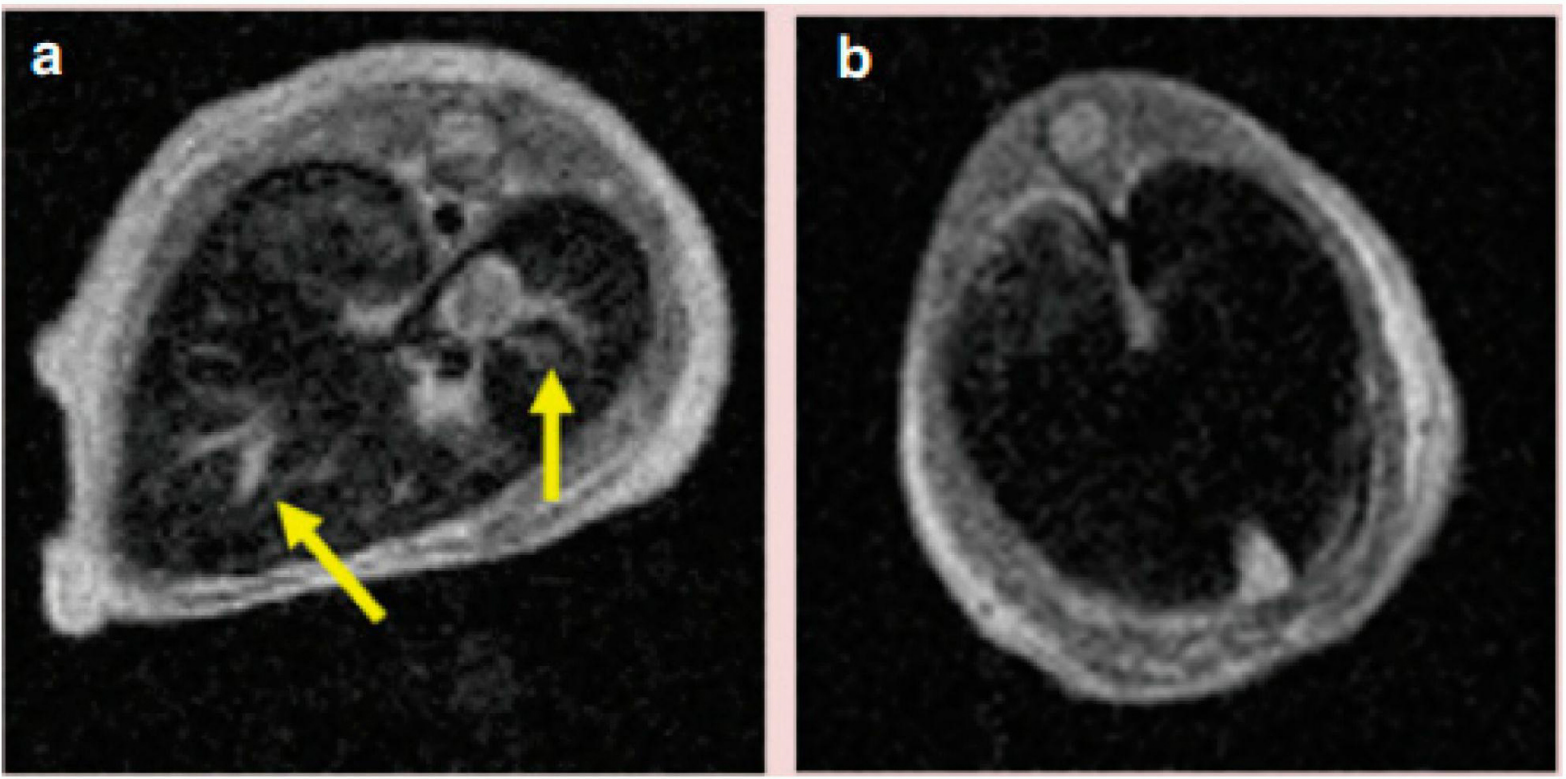
**T2-weighted MRI of hepatic contrast enhancement in a murine model**. Axial T2-weighted MRI images of mouse liver acquired 60 minutes after administration of (a) raspberry-shaped superparamagnetic iron oxide nanoparticles and (b) the commercially available agent ferucarbotran®. Enhanced visualization of hepatic vasculature is observed with nanoparticle-based contrast as indicated by the arrows in (a). Reproduced with permission by MDPI, Inc [35].

Superparamagnetic iron oxide nanoparticles (SPIONs) have emerged as promising alternatives to gadolinium-based contrast agents. These offer high relaxivity and the ability to target specific cellular or molecular processes. As seen in Fig. 3 (Ref. [35]), SPIONs have great utility in visualizing hepatic vasculature in mice, which is not otherwise observed with a commercially available contrast agent. Additionally, SPION accumulation in areas of macrophage infiltration, such as in inflamed or infarcted myocardium, provides enhanced contrast in detecting early myocardial injury or inflammation [35].

SPIONs can be engineered for longer circulation times and for targeted delivery, enabling molecular imaging of atherosclerosis and myocarditis. Another article highlights how functionalization of manganese-based contrast agents enables their use in theranostics, combining diagnostic imaging with targeted therapy [36].

Additionally, Al-Shimmari et al. [37] describe advances in nanoparticle coatings to contrast agents for enhanced biocompatible cardiovascular tissue uptake. This innovation results in contrast agents that serve a more personalized and customizable approach to contrast uptake for CMR.

#### 2.4.2 Discussion of Safety Concerns Surrounding Gadolinium-Based Agents and Alternatives

GBCAs have been the cornerstone of cardiac MRI enhancement, particularly for LGE imaging. For safety concerns, the ACR manual of contrast media emphasizes NSF as a serious documented adverse outcome associated with GBCA use. The manual acknowledges the phenomenon of gadolinium retention and deposition in tissues such as brain, bone, and other organs after multiple doses, even in patients with normal renal function, although the clinical significance of tissue retention remains unclear. Lastly, hypersensitivity and physiologic reactions to GBCAs are discussed as a safety concern, although infrequent [36]. This has led to growing scrutiny and a push toward safer alternatives [38].

Per the ACR manual, the diagnostic benefit of GBCA-enhanced imaging should be weighed against the rare but potentially serious safety concern. The 2021 ACR-National Kidney Foundation (NKF) guidelines give preferential selection to Group II or Group III agents, particularly in patients with impaired renal function, due to their very low risk of NSF. In patients with an estimated glomerular filtration rate (eGFR) below 30 mL/min/1.73 m^2^, the consensus notes that the NSF risk associated with Group II agents is extremely low and that delaying clinically indicated contrast-enhanced MRI may confer greater harm than proceeding with contrast administration. The statements further advise against routine initiation or modification of dialysis solely for GBCA removal and emphasize that patient counseling regarding potential risks should be guided by clinical context [34].

While macrocyclic GBCAs, a more stable chemical GBCA, are associated with lower retention compared to linear agents, ongoing efforts continue to explore contrast-free imaging techniques and non-gadolinium agents. Poggiarelli et al. [39] emphasize that manganese-based alternatives show promise in preclinical studies as emerging agents but require further validation for routine clinical use.

#### 2.4.3 Discussion on Non-Contrast MRI Techniques

These safety concerns have reshaped research priorities in CMR by encouraging exploration and development of non-contrast techniques and safer, more targeted contrast agents. Innovative non‑contrast approaches are emerging, including advanced parametric mapping strategies for tissue characterization such as myocardial T1‑rho mapping, cine imaging to capture dynamic or moving images of the heart throughout the cardiac cycle, and texture analysis to quantify the spatial patterns, heterogeneity, and relationships between pixel intensities in an image. These protocols can help differentiate etiologies of heart failure by quantifying myocardial characteristics such as relaxation times and structural heterogeneity, offering quantitative diagnostics independent of contrast enhancement [40]. Emerging parametric mapping strategies and quantitative image analysis further enhance the sensitivity of non-contrast MRI, suggesting it could serve as a first-line or adjunctive tool, reduce scan time, minimize patient risk, and support longitudinal monitoring.

### 2.5 Artificial Intelligence and Machine Learning in Cardiac MRI

#### 2.5.1 Role of AI in Image Reconstruction and Noise Reduction

AI is currently being integrated into CMR to improve image quality and streamline acquisition. Significant noise and artifact reduction has been achieved through deep learning-based reconstruction algorithms. This produces high-quality images even from undersampled data. These algorithmic models also allow for faster acquisitions without sacrificing diagnostic accuracy [41].

Morales et al. [42] demonstrate how AI-assisted reconstruction decreases motion artifact, offers real-time image availability, and reduces technician-dependent post-processing. AI-assisted reconstruction specifically addresses noise reduction, motion correction, and artifact suppression. This aids in free breathing and real-time imaging protocols. Another advancement includes AI-based methods used to standardize the interpretation of CMR by automating measurements of ejection fraction, myocardial mass, and strain. This increased the consistency in diagnostic accuracy across institutions.

AI’s ability to reduce noise, reconstruct images, and aid in data interpretation in CMR, especially in settings with limited expert availability or when rapid decision-making is critical, makes CMR safer, faster, and more accurate.

#### 2.5.2 Machine Learning Models for Automated Segmentation, Disease Classification, and Data Interpretation

Machine learning models are changing CMR interpretation by automating segmentation and clinical decision support. Additionally, convolutional neural networks (CNNs), an AI neural network for visual data analysis, have shown somewhat promising results for cardiac structure segmentation. This includes segmentation of the tissues and myocardial structures in various pathologies using a variety of contrasts and pathologies [43].

Several studies have been performed recently with a commonly used CNN called U-Net to determine agreeability between automated and manual segmentations using the Dice index, with a higher score indicating segmentation results comparable to those of human experts. The majority of these studies using this AI model produced a Dice score >85% for left ventricle (LV) segmentation, which can significantly reduce time and inter-observer variability in volumetric and functional analysis [44]. However, even with these recent improvements, certain limitations remain, with many of these algorithms showing difficulty in appropriate segmentation of basal and apical regions. Additionally, multiple such algorithms utilized single-center databases for training purposes, which may lead to a deficiency in generalizability of the results.

A more recent study performed by Hadamitzky et al. [45] used separate AI models for myocardial segmentation using different MRI sequences and for disease classification of various pathologies, both of which were evaluated using a Dice score and Area Under the Receiver Operating Characteristic Curve (AUC), respectively. This study demonstrated favorable results pertaining to accurate segmentation with an average Dice score of 0.89 amongst various MRI sequences [45]. However, there was significant variability for disease classification with AUC scores >0.90 for various cardiomyopathies and cardiac amyloidosis but less than desirable results with old myocarditis (AUC 0.68), acute myocarditis (AUC 0.63), and acute myocardial infarction (AUC 0.51) [45].

Additional advancements have been made in regard to AI interpretation of CMR for screening and diagnostic purposes of various cardiovascular diseases. Wang et al. [46] performed a large-scale study that utilized two AI models for imaging interpretation. The results showed excellent performance by the AI models, demonstrating highly accurate screening and diagnostic findings for cardiovascular disease with AUC of 0.988 and 0.991, respectively [46].

#### 2.5.3 Challenges and Ethical Issues Associated With Use of AI in CMR

Although AI models have shown promising results with integration into CMR, many challenges and ethical issues remain with the development and use of these AI models. For example, high computational costs remain a significant challenge in developing accurate AI algorithms, which can subsequently limit access in regions with limited resources to implement the newly developed AI technology [47]. Additionally, an important ethical issue regarding patient data privacy will continue to play a significant role in the development and implementation of these AI models given the necessity of large datasets to appropriately train the algorithms [47].

Another major point of contention comes into play when considering the inherent biases involved with the use of AI models. The development of these AI models is highly reliant on the quality of the datasets used for their training purposes, which may create bias against underrepresented populations that were largely excluded from these datasets [48]. Utilization of such AI algorithms can produce false interpretive results for these populations and lead to inappropriate treatment guidance. Therefore, it is imperative that the AI models in consideration of implementation are appropriately trained using datasets that include underrepresented populations in order to provide the most accurate results for all types of patient populations [49].

Finally, another major hurdle to consider as it pertains to the use of AI models is the willingness to adopt this technology by the hospitals and physicians, as well as taking into consideration the general comfort by the patients in trusting AI to make decisions in their patient care [47]. There has been growing use of large language models (LLM) for various tasks such as summarizing clinical information, creating succinct impressions, and reorganizing text. However, these models have also proved to be faulty at times, with the most considerable drawback being the generation of false information or findings. While automation of report impressions is more widely used now, many other LLMs still require further development [50]. Overall, improved development of AI models in conjunction with further education at multiple levels of the healthcare system is required to decrease the resistance to implementation of these models in order to achieve the benefits provided by the constantly evolving AI algorithms.

**Fig. 4.**
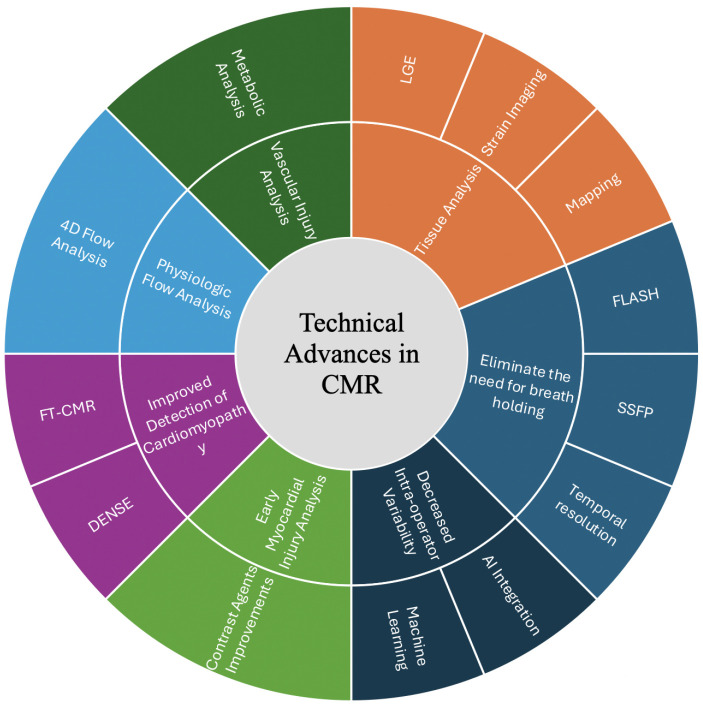
**Key technological advances in cardiovascular magnetic resonance imaging**. Summary of recent technical developments in CMR, including improvements in temporal resolution, steady-state free precession (SSFP) and Fast Low-Angle Shot (FLASH) sequences, parametric mapping, strain imaging, late gadolinium enhancement (LGE), 4D flow analysis, feature-tracking and displacement encoding with stimulated echoes (DENSE) techniques, metabolic imaging, contrast agent development, and integration of machine learning and artificial intelligence to improve diagnostic performance and workflow efficiency. FT-CMR, Feature Tracking Cardiovascular Magnetic Resonance.

Fig. 4 below summarizes technical advances in CMR as they have been discussed so far.

## 3. Clinical Applications of CMR

### 3.1 Cardiomyopathies

CMR has become a cornerstone in evaluating hypertrophic cardiomyopathy, particularly for its ability to detect myocardial fibrosis through LGE. In a comprehensive study involving 1423 patients, Mentias et al. [51] found that the presence of LGE was associated with more than a twofold increase in the risk of sudden cardiac death. Notably, for every 10% increase in LGE, there was a corresponding 36% rise in the risk of sudden cardiac death, underscoring the prognostic significance of LGE extent in these patients.

Emphasizing the clinical value of precise fibrosis measurement, Aquaro et al. [52] showed that patients with LGE reaching or exceeding 15% of left ventricular mass experienced significantly higher rates of life-threatening cardiac events, including sudden cardiac death and appropriate defibrillator therapy. In their prospective study of 105 patients followed over a median of 52 months, the proportion of individuals exceeding this threshold rose from 9% to 20%, changes closely linked to a sharp rise in adverse outcomes. The researchers also identified that a rate of LGE progression greater than 0.07 grams per month was the strongest independent predictor of these events, enhancing risk stratification beyond a single LGE measurement [52].

Beyond LGE, emerging techniques like T1 mapping have enhanced the detection of diffuse myocardial fibrosis, even in the absence of LGE. This quantitative approach provides early diagnostic and prognostic information, aiding in risk stratification and management decisions.

In the context of dilated cardiomyopathy, CMR serves as the gold standard for evaluating ventricular volumes, function, and myocardial tissue characterization. Mid-wall LGE patterns are characteristic of non-ischemic dilated cardiomyopathy and have been linked to increased risks of heart failure hospitalization and mortality. Halliday et al. [53] conducted a study involving 472 patients with non-ischemic dilated cardiomyopathy and found that the presence of mid-wall LGE was associated with a significant increase in the risk of sudden cardiac death. Specifically, patients with mid-wall LGE had a 5-year sudden cardiac death rate of 12.3%, compared to 2.3% in those without LGE. Importantly, this association remained significant even after adjusting for left ventricular ejection fraction, highlighting the independent prognostic value of mid-wall LGE in dilated cardiomyopathy.

Additionally, the extent and location of LGE have been shown to influence outcomes. A study by Halliday et al. [54] demonstrated that patients with extensive LGE (greater than 5% of left ventricular mass) had higher rates of heart failure hospitalization and all-cause mortality. Furthermore, septal LGE was particularly associated with arrhythmic events, suggesting that both the amount and distribution of fibrosis are critical factors in risk assessment.

CMR plays a pivotal role in diagnosing and assessing cardiac amyloidosis, a form of restrictive cardiomyopathy. T1 mapping and extracellular volume quantification have emerged as sensitive techniques for detecting diffuse myocardial infiltration. Fontana et al. [55] investigated the prognostic value of native T1 and extracellular volume in patients with systemic light-chain amyloidosis. They found that elevated native T1 and extracellular volume values were strong predictors of mortality, independent of traditional biomarkers. Specifically, patients with extracellular volume values above 0.45 had a median survival of 22 months, compared to 62 months in those with lower extracellular volume values [55].

Moreover, LGE patterns in cardiac amyloidosis are distinctive, often presenting as global subendocardial or transmural enhancement. These patterns, combined with quantitative mapping techniques, enhance diagnostic accuracy and provide valuable prognostic information. In a study by Banypersad et al. [56], extracellular volume had a higher diagnostic odds ratio for assessing cardiac amyloidosis than LGE and a higher hazard ratio for adverse events compared with LGE and native T1, hitting superior prognostic utility.

Advanced CMR techniques, such as T1 and T2 mapping, offer quantitative assessments of myocardial tissue characteristics. T1 mapping is particularly useful for detecting diffuse fibrosis, while T2 mapping is sensitive to myocardial edema. O’Brien et al. (2022) [57] conducted a comprehensive review highlighting the clinical applications of T2 mapping in myocardial diseases. They emphasized that elevated T2 relaxation times are indicative of increased myocardial water content and can serve as early markers of reversible myocardial injury [57]. Importantly, T2 mapping has demonstrated utility in diagnosing and monitoring conditions like myocarditis, acute myocardial infarction, and transplant rejection [57].

Furthermore, the integration of T1 and T2 mapping techniques enhances the ability to characterize myocardial tissue comprehensively, facilitating early diagnosis, risk stratification, and monitoring of therapeutic responses across various cardiomyopathies.

### 3.2 Ischemic Heart Disease

CMR has become an indispensable tool in evaluating myocardial infarction and assessing myocardial viability. The technique of delayed enhancement results in precise delineation of infarcted tissue. In one landmark clinical review, segments with fibrosis covering 50% or more of the myocardial wall were classified as non‑viable, while those with under 50% fibrosis retained potential for functional recovery after revascularization [58].

CMR delivers a comprehensive, noninvasive evaluation of ischemic cardiomyopathy by combining precise measurement of heart function, detailed tissue characterization, and assessment of blood flow [59]. Functional imaging accurately measures ventricular volumes and ejection fraction, T1 and T2 mapping detects fibrosis and edema, and first-pass perfusion imaging identifies ischemic regions under stress [59]. This integrated approach supports informed decision-making around revascularization strategies in ischemic heart disease without exposing patients to radiation, enhancing both diagnostic clarity and patient safety.

Myocardial perfusion imaging with CMR offers a noninvasive and highly accurate means of detecting ischemic coronary artery disease. In a systematic review and meta-analysis of 14 studies encompassing 650 patients and 1073 coronary arteries, Li et al. [60] reported a pooled patient-level sensitivity of 90% (95% CI: 86–93%) and specificity of 87% (95% CI: 82–90%) for stress perfusion CMR—using invasive fractional flow reserve as the reference standard. At the territory level, sensitivity and specificity were 89% (95% CI: 83–92%) and 86% (95% CI: 77–92%), respectively. These high accuracy figures emphasize CMR perfusion’s reliability in identifying true ischemia compared to invasive measurements [60,61].

CMR demonstrates high diagnostic accuracy for detecting coronary artery disease, matching the performance of positron emission tomography and surpassing single-photon emission computed tomography. In a meta-analysis of 28 studies with fractional flow reserve as the reference, Yang et al. [61] reported a pooled sensitivity of 88% (95% CI: 80–93%) and specificity of 89% (95% CI: 85–93%) for stress perfusion CMR—identical to positron emission tomography (PET) and significantly higher than single photon emission computed tomography (SPECT) (69% sensitivity). The superior spatial resolution and lack of radiation exposure make CMR a robust tool for both anatomical and functional assessment in patients with suspected or known coronary artery disease [61].

#### Myocardial Infarction With Nonobstructive Coronary Arteries (MINOCA)

CMR provides high-resolution, radiation-free tissue characterization and allows accurate assessment of wall motion, edema, microvascular obstruction, and myocardial viability [62]. The use of CMR has been emphasized in the evaluation of patients with suspected MINOCA, which is defined by clinical evidence of myocardial infarction in the absence of obstructive coronary artery disease. The primary role of CMR is to distinguish true ischemic myocardial infarction from non-ischemic causes of myocardial injury, thereby identifying conditions such as myocarditis and Takotsubo syndrome, which frequently mimic myocardial infarction at presentation and can be reliably differentiated using CMR [63]. An abundance of studies show high diagnostic yield with CMR in those with suspected MINOCA. All in all, about 20% of the patients were found to have true MI, about 33% were found to have myocarditis, and an even smaller proportion of patients were diagnosed with Takotsubo syndrome [63].

Studies show that diagnostic yield decreases as time from acute event increases. Dastidar et al. [64] conducted a retrospective analysis comparing early CMR (within 2 weeks) with delayed CMR (>2 weeks) and found that performing CMR earlier substantially increased its diagnostic yield, rising from 51% to 88%. Early imaging was also associated with a greater clinical impact, with changes in diagnosis and patient management increasing from 51% to 76% [64].

CMR techniques for MINOCA include early and LGE. Use of early gadolinium enhancement (EGE) has declined due to inconsistent image quality, with data suggesting that omission of EGE did not significantly reduce diagnostic accuracy. LGE remains central for tissue characterization, with phase-sensitive inversion recovery (PSIR) preferred over magnitude inversion recovery because of its reduced sensitivity to inversion time selection. Multiparametric mapping, incorporating T1 mapping, T2 mapping, and ECV assessment, has demonstrated useful diagnostic value in myocarditis, but its role in MINOCA has not been systematically studied [63].

A Chinese retrospective study on 51 patients diagnosed with MI, of which 21 met diagnostic criteria of MINOCA per European Society of Cardiology (ESC) guidelines and 30 met criteria for myocardial infarction with coronary artery disease (MICAD), was performed [62]. All patients underwent CMR, and myocardial infarct segments were identified using LGE imaging. Baseline characteristics, including body mass index (BMI), sex, and coronary risk factors such as hypertension, diabetes, obesity, and coronary heart disease, were recorded. The MINOCA group was significantly younger, with lower rates of dyslipidemia and fewer smokers compared with the MICAD group, although the latter was not statistically significant [62]. Lastly, CMR-related findings from this Chinese cohort suggested MINOCA patients had smaller MI areas, lower scores of transmural extent, and fewer affected segments with left ventricular function preservation relative to MICAD patients, which may help stratify patient risk and guide individualized management in MINOCA [62]. These CMR findings help characterize the pattern of MINOCA and can assist in clinical decision-making and in identifying underlying mechanisms.

### 3.3 Congenital Heart Disease

CMR has become an indispensable tool in the evaluation of congenital heart disease, offering detailed anatomical and functional information crucial for presurgical planning. Advancements in CMR technology have led to improved spatial and temporal resolution, enabling more accurate visualization of complex cardiac structures [65].

One significant advancement is the development of four-dimensional flow magnetic resonance imaging, which enables a comprehensive assessment of blood flow dynamics within the heart and great vessels. This technique provides valuable insights into hemodynamic parameters, aiding in the assessment of conditions such as valve regurgitation and shunt quantification [65].

Furthermore, CMR has proven effective in the postoperative evaluation of congenital heart disease patients. A study by Saraya et al. [66] demonstrated that CMR effectively identified postoperative complications, such as pulmonary branch stenosis and right ventricular failure, in patients who had undergone surgical repair for tetralogy of Fallot. This highlights the role of CMR in both pre- and post-surgical assessment, facilitating timely interventions when necessary.

### 3.4 Cardiac Inflammation and Infection

The utility of CMR extends to the evaluation of post-viral cardiac inflammation, a condition gaining prominence in the context of recent viral pandemics. CMR can detect residual myocardial inflammation, fibrosis, or scarring that may persist after the acute phase of viral infections, such as COVID-19. These findings have significant prognostic implications, as persistent myocardial involvement can lead to long-term cardiac dysfunction [67].

Early detection through CMR allows for timely intervention and monitoring, potentially mitigating adverse outcomes. Moreover, the noninvasive nature of CMR makes it an ideal modality for serial assessments in patients recovering from viral myocarditis or pericarditis [67].

### 3.5 Cardiac Arrhythmias

CMR is essential for evaluating arrhythmias by uncovering myocardial scar and structural abnormalities that support arrhythmogenic substrates. LGE sequences reliably detect myocardial fibrosis, a key trigger for reentrant ventricular tachycardias [68]. In arrhythmogenic right ventricular cardiomyopathy, CMR reveals hallmark features including right ventricular dilation, regional wall motion abnormalities, and fibrofatty infiltration. In a cohort of 140 confirmed arrhythmogenic right ventricular cardiomyopathy (ARVC) patients, Aquaro et al. [68] found that CMR-detected left ventricular involvement (fibrosis or fat) independently predicted major cardiac events (hazard ratio 3.69, 95% CI 1.57–8.65), and abnormal CMR findings were present in 90% of patients with events, yielding a negative predictive value of 97%. Even in patients who were only partially satisfied with the traditional 2010 Task Force Criteria, fibrofatty changes appeared frequently on CMR, indicating early disease stages [69]. These insights highlight CMR’s critical role in both diagnosis and risk stratification in ARVC.

Expanding on this point, CMR provides a comprehensive assessment of ventricular volumes and function, aiding in the identification of patients at increased risk of ventricular arrhythmias. The integration of functional and tissue characterization data enhances the understanding of arrhythmogenic substrates, facilitating more accurate diagnosis and management strategies [68].

The utilization of CMR extends to the planning and guidance of catheter ablation procedures. By delineating the extent and distribution of myocardial scar tissue, CMR assists in identifying arrhythmogenic foci and tailoring ablation strategies accordingly. In a prospective cohort study by Soto-Iglesias et al. [70], CMR-guided ablation reduced procedure duration (107 ± 59 min vs. ~205 min), fluoroscopy time (10 ± 4 min vs. ~23 min), and radiofrequency ablation time. Furthermore, ventricular tachycardia inducibility post-ablation decreased significantly compared to non-CMR-guided procedures, and rates of recurrence at 12-month follow-up were lower [70]. This evidence highlights the value of MRI-guided catheter ablation in improving efficiency and clinical outcomes in ventricular tachycardia management.

In addition, CMR can be employed post-ablation to assess lesion formation and detect potential gaps in ablation lines, thereby informing the need for additional interventions. The non-invasive nature and high spatial resolution of CMR make it an invaluable modality in both the pre-procedural planning and post-procedural evaluation of catheter ablation therapies [71].

## 4. Quantification and Cardiac Biomarkers

### 4.1 Tissue Characterization

CMR has progressed from qualitative LGE imaging to quantitative parametric mapping, enabling voxel-wise measurement of tissue relaxation times and thus a “virtual biopsy” of the myocardium. By directly indexing fibrosis (T1/ECV), edema (T2), and iron (T2*), these techniques overcome the binary limitations of LGE and allow diffuse disease to be detected long before wall motion deteriorates.

Native T1 and its derivative, ECV, now represent the leading non-invasive markers of interstitial expansion. In a biopsy-validated dilated cardiomyopathy (DCM) cohort, Nakamori et al. [72] demonstrated strong correlations between native T1 (r = 0.77) and ECV (r = 0.86) with collagen volume fraction, confirming CMR as a reliable surrogate for histology. Prognostically, the International T1 Multicentre CMR Outcome Study showed that every 20-ms increment in native T1 increased the hazard of death or heart failure admission by 10% among non-ischemic cardiomyopathy patients [73]. Extending these findings to an unselected population, Raisi-Estabragh et al. [74] analyzed 42,308 UK Biobank participants and found that higher native T1 independently predicted incident atrial fibrillation, heart failure, and all-cause mortality, underscoring its utility for population-level risk profiling.

Quantitative T2 captures absolute myocardial water content and hence active injury. In suspected acute myocarditis, Spieker et al. [75] reported that global T2 >60 ms conferred a six-fold higher risk of major adverse cardiac events at 12 months compared with normal T2, even after adjusting for LGE. Mapping, therefore, satisfies the revised Lake Louise criteria, providing reproducible thresholds for diagnosing and monitoring inflammatory cardiomyopathies. A 2022 JCMR review of >50 studies by O’Brien et al. [57], confirmed T2 mapping’s incremental diagnostic yield across myocarditis, infarction, transplant rejection, and dilated cardiomyopathy, and emphasized its use for longitudinal therapy monitoring.

Myocardial T2* is uniquely sensitive to paramagnetic iron and has transformed the management of transfusion-dependent anemias. In a 2019–2021 Iranian cohort of 67 β-thalassemia major patients, Zavar et al. [76] showed that T2* values <20 ms reliably identified subclinical cardiac siderosis and correlated with arterial elasticity indices, guiding early chelation before ejection fraction declines. Recent TIF guidelines now recommend annual T2* measurements, crediting the technique with halving cardiac mortality in thalassemia through chelation intensification when T2* falls below 10 ms.

Combining mapping with LGE yields a truly multiparametric assessment: LGE delineates focal replacement scar, while elevated T1 or ECV unmasks diffuse fibrosis in “normal” myocardium, and T2/T2* identifies superimposed edema or iron. In DCM, patients without LGE but with diffusely elevated ECV still show impaired strain and worse outcomes, whereas the coexistence of LGE and high ECV portends the poorest prognosis, advocating for multiparametric imaging to fully stage disease [73]. Overall, parametric mapping now supplies reproducible, tissue-specific biomarkers that complement LGE, fulfill contemporary diagnostic criteria, and enable serial quantification of therapeutic response.

### 4.2 CMR for Risk Stratification

DCM, a common non-ischemic cause of heart failure, exemplifies how CMR-detected myocardial fibrosis can serve as a powerful prognostic marker. Halliday et al. [54] studied 874 DCM patients and found that even a small area of septal LGE (<5% of left ventricular mass) doubled the five-year risk of all-cause mortality or sudden cardiac death (SCD), underscoring that even minimal scar can significantly elevate risk. Similarly, an earlier study focusing on patients with milder DCM (left ventricular ejection fraction (LVEF) 40–55%) showed that mid-wall LGE was associated with a five-fold higher incidence of SCD, reinforcing the prognostic impact of myocardial fibrosis across the spectrum of disease severity [53]. Moreover, beyond these focal scar findings, diffuse fibrosis measured by T1 mapping provides independent prognostic information. In the same cohort, Puntmann et al. [73] demonstrated that an elevated native T1 value, which reflects diffuse interstitial fibrosis, predicted higher mortality risk even after accounting for LGE presence and left ventricular ejection fraction (EF). Collectively, these findings have spurred proposals to integrate CMR-derived fibrosis metrics into implantable cardioverter-defibrillator (ICD) selection algorithms, particularly for patients whose EF alone would not conventionally qualify them for device therapy.

In hypertrophic cardiomyopathy (HCM), similarly, the extent of myocardial fibrosis detected by LGE is a key determinant of arrhythmic risk. A meta-analysis of 2993 HCM patients showed that LGE involving more than 15% of left ventricular mass was associated with an approximately threefold increase in SCD risk (odds ratio ~3.4) [77]. Importantly, this elevated risk was independent of other established risk factors such as pronounced maximal wall thickness, a family history of SCD, or episodes of unexplained syncope. As a result, quantitative scar assessment by CMR has been added to contemporary HCM risk stratification models to refine risk estimates. This practice helps guide prophylactic ICD implantation in borderline cases where traditional risk factors alone provide an equivocal risk assessment.

In coronary artery disease (CAD), by contrast, the prognostic utility of CMR lies in its ability to quantify inducible ischemia while simultaneously assessing scar from prior infarctions in a single noninvasive exam. Stress perfusion CMR provides a comprehensive assessment by measuring ischemic burden and infarct scar together, and this approach has proven valuable for risk stratification in patients with suspected CAD. For instance, Sammut et al. [78] evaluated 395 patients with suspected CAD and found that incorporating a quantitative myocardial perfusion reserve measure from CMR into a standard clinical risk model significantly improved prognostic discrimination. This added imaging metric reclassified about 16% of patients into more appropriate risk categories and identified a subset with annual major adverse cardiac event (MACE) rates exceeding 4%, identifying a high-risk group who would benefit from more aggressive management [78]. Equally important, a normal stress CMR result portended a very low risk (under 1% per year) of cardiac events, supporting the safe deferral of invasive coronary angiography in such low-risk individuals.

Beyond the more common cardiomyopathies, advanced CMR tissue mapping techniques also offer critical prognostic insight in rarer infiltrative diseases like cardiac amyloidosis. A 2024 systematic review encompassing 16 studies and a total of 1356 patients demonstrated strong associations between CMR mapping parameters and outcomes in amyloidosis [79]. Specifically, each 3% increase in myocardial ECV was associated with roughly a 16% increase in mortality risk, and every 60-millisecond prolongation of native T1 corresponded to about a 33% increase in mortality [79]. These quantitative imaging thresholds have become clinically meaningful benchmarks and are now being used to guide the timing of interventions in light-chain (AL) amyloidosis. For instance, a patient with markedly elevated ECV or native T1 may prompt earlier initiation of chemotherapy or referral for cardiac transplant evaluation, ensuring that therapy is escalated before the disease progresses to an irreversible stage.

Finally, integrating CMR findings with traditional clinical risk scores can substantially refine patient risk stratification, enabling more personalized management. Even patients deemed low-risk by conventional criteria may be up-classified as high-risk if CMR reveals occult abnormalities. For example, an incidental myocardial scar on LGE or an elevated native T1 suggesting diffuse fibrosis can prompt a reassessment of prognosis. Large cohort data support this reclassification approach [74]. In the UK Biobank study, adding native T1 mapping values to a predictive model for incident heart failure improved the model’s discrimination, raising the C-statistic from 0.78 to 0.83 compared to a model using demographic and biomarker data alone [74]. Similarly, in DCM, incorporating LGE findings into contemporary risk models has been shown to adjust risk categories substantially: over 30% of patients who would have qualified for an ICD based on low EF or other clinical criteria could be downgraded to a lower-risk category if no scar is present (potentially sparing them an unnecessary device), whereas approximately 15% of patients previously considered safe were upgraded to higher-risk status upon detection of scar tissue [74]. Use of CMR supports prognostic assessment, allowing for individualized risk evaluation. This guides clinicians regarding decisions about ICD placement, early initiation of guideline-directed medical therapy (GDMT) for high-risk patients, and prompt referrals if needed.

## 5. Challenges and Limitations of CMR

With its noninvasive approach and wide-ranging clinical utility, cardiac MRI has firmly established itself as a key diagnostic tool in the field of cardiovascular medicine. Despite the growing role of CMR in cardiovascular diagnostics, various limitations continue to impede its broader clinical utilization. To ensure that CMR can effectively improve patient care, it is crucial to recognize and address its limitations through continued technological innovation, enhanced accessibility, and targeted clinical training. Ultimately, optimizing the use of CMR has the potential to significantly enhance diagnostic precision and improve outcomes in patients with a broad spectrum of cardiovascular diseases.

### 5.1 Patient-Related and Physiological Limitations

#### 5.1.1 Psychological and Physiological Barriers

While CMR is a non-invasive imaging modality that poses no known ionizing radiation risk to patients or caregivers, its effective implementation in clinical practice is often challenged by patient-related limitations. These limitations include anxiety, claustrophobia, the presence of medical implants and devices, adverse reactions to contrast agents, and potential risks during pregnancy [80]. Claustrophobia and lack of cooperation can significantly impede image acquisition, often necessitating sedation to facilitate a complete and diagnostically useful examination [81].

#### 5.1.2 Motion and Physiological Challenges

Beyond these psychological and physiological barriers, mechanical challenges related to patient and organ motion can also compromise image quality and efficiency. In addition to these limitations, a major constraint includes both voluntary patient motion and physiological motion associated with cardiac and respiratory activity. Even minor movements can result in motion artifacts, which reduce image quality and often require extended scan protocols [82]. High-quality CMR images require patients to remain still throughout the entire scan, which can be very difficult in certain patient populations, such as pediatric patients or those experiencing pain, anxiety, or claustrophobia.

The challenge of motion is magnified in patients with specific physiological limitations and gets further exacerbated in individuals with irregular heart rhythms or limited breath-holding capacity [29,82,83]. Arrhythmias disrupt cardiac gating, while breath-hold failure can prolong scan times and diminish diagnostic accuracy [29,82,83]. Physiological motion caused by the beating heart and respiratory cycles further complicates image acquisition, particularly when standard ECG gating and breath-hold techniques are insufficient or fail due to arrhythmias or inconsistent breathing patterns. These factors can lead to misregistration, blurring, and ghosting, which significantly compromise spatial and temporal resolution [82,84].

#### 5.1.3 Mitigation Strategies

To overcome these barriers, various motion mitigation strategies have been developed to enhance image fidelity. Advanced motion mitigation strategies like navigator gating, parallel imaging, and free-breathing real-time techniques address some of these limitations, but they often introduce an added complexity, prolong scan duration, or require specialized reconstruction algorithms and models [27,28]. As these techniques may not be readily available or feasible in all clinical settings, they also highlight disparities in accessibility. This proves the need for individualized patient assessment prior to CMR, as well as the continued development of tailored protocols and technological adaptations to improve patient tolerability and diagnostic performance of the MRI.

### 5.2 Access and Geographic Availability

Despite its growing clinical importance, CMR is limited by several technological and operational constraints. Although essential imaging hardware and expertise are available at many medical centers, their accessibility is variable and often dictated by geographical distribution and institutional capacity. For instance, a recent analysis of U.S. data revealed substantial regional disparities in cardiac MRI availability, ranging from 4.4 to 52.6 centers per million Medicare beneficiaries in 2018, suggesting that access is closely tied to location and institutional experience [85]. Furthermore, more robust CMR programs are predominantly at academic healthcare institutions, where most of the studies are performed by cardiologists and radiologists [85,86]. This reinforces the relationship between CMR capacity as well as the dependence on specialized personnel.

### 5.3 Cost and Infrastructure Constraints

In addition to expertise requirements, high installation and operation costs further constrain the adoption of CMR technology. Modern MRI machines can cost upwards of $ 1 million each, not including the additional infrastructure, maintenance, and power demands required of an institution [87]. This financial burden disproportionately affects low- and middle-income countries where geographical access, education and training for technical staff and power availability are scarce [87,88]. Even in high-income countries, significant health system expenditures can deter institutions from expanding CMR capacity and availability [87,88].

These technological and operational challenges contribute heavily to the underutilization of CMR, despite its well-established clinical value. To bridge this gap, targeted strategies must prioritize expanding access to CMR infrastructure, promoting equitable geographic access, prioritizing workforce development, and creating sustainable cost-effective strategies. Addressing these limitations and challenges will enhance the integration of CMR into routine cardiovascular diagnostics and ensure that its benefits are more broadly realized across the healthcare system.

### 5.4 Training and Workforce Limitations

Even when imaging systems are in place, their effective clinical utilization relies on the availability of adequately trained professionals. The successful completion of CMR studies requires not only technical expertise and proficiency in scan acquisition but also interpretive expertise. Despite the growing advancements in CMR as well as increasing clinical demand in North America, the number of radiology and cardiology trainees that pursue specialized CMR training remains limited [85,86]. While CMR offers significant career opportunities for these physicians, challenges persist in accessing adequate training and establishing a professional focus in this field [86,89]. Strategic efforts to support the professional development of practitioners are critical to advancing and promoting the widespread adoption and improved accessibility of CMR.

### 5.5 Contrast Agent Safety

#### 5.5.1 Gadolinium Safety and NSF

The use of contrast agents is integral to many CMR protocols and examinations, significantly enhancing table diagnostic accuracy. Despite their critical role in CMR, the use of GBCAs presents several limitations and challenges. GBCAs are essential for evaluating myocardial fibrosis, viability, and perfusion through techniques such as LGE [90,91]. However, the administration of GBCAs requires careful consideration within various patient populations and is dependent on individual patient characteristics.

**Fig. 5.**
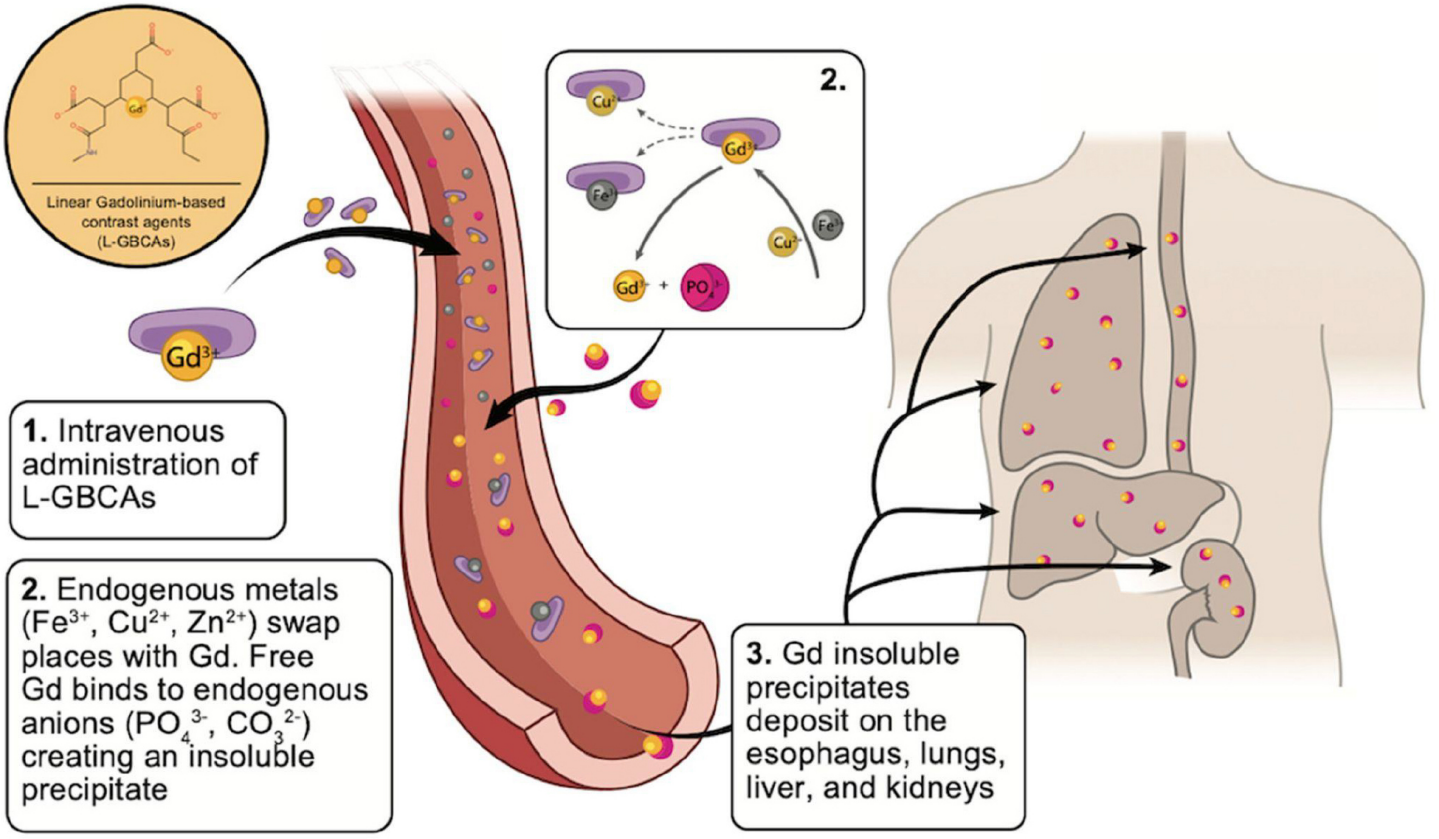
**Pathophysiological mechanism of NSF**. Schematic representation of gadolinium release from linear gadolinium-based contrast agents, subsequent binding to anions, formation of insoluble complexes, and deposition in multiple organs, contributing to the development of NSF. Reproduced with permission by MDPI, Inc [94]. Gd, gadolinium; L-GBCAs, linear gadolinium-based contrast agents.

One of the primary concerns is the risk of NSF, a rare but serious condition associated with GBCA exposure in patients with severe renal impairment (eGFR ≤30 mL/min/1.73 m^2^) [90,92,93]. Though the pathophysiology is not entirely understood, it is believed that de-chelation of gadolinium (Gd) from a linear-gadolinium-based contrast agent (L-GBCA) and subsequent binding of free Gd with anions produce insoluble precipitates, as seen in Fig. 5 (Ref. [94]). These insoluble precipitates can then deposit in multiple organ systems and stimulate the release of pro-inflammatory and pro-fibrotic cytokines, which induce the signs & symptoms seen in NSF [94]. While the introduction of more stable macrocyclic agents has substantially reduced the incidence of NSF, it has not eliminated the risk entirely [34,93]. As a result, routine assessment of renal function remains a critical step before administering contrast agents [34,93].

#### 5.5.2 Gadolinium Deposition and Regulatory Considerations

Another emerging issue involves gadolinium deposition in tissues such as the brain (dentate nucleus and globus pallidus), bone, and skin, even in patients with normal renal function [92,95,96,97,98]. Repeated administration of GBCAs, especially the linear agents, has been associated with increased deposition and retention [92]. While the exact biological mechanism and long-term clinical consequences remain unclear, these findings have prompted heightened regulatory attention and scrutiny. The US Food and Drug Administration (FDA) has issued recommendations to limit the use of linear GBCAs and promote informed patient consent and risk-benefit assessment prior to administration [96].

#### 5.5.3 Non-Contrast Techniques

Collectively, these limitations underscore the importance of patient screening, an individualized risk-benefit assessment, and continued research into alternative imaging strategies. The development of non-contrast CMR techniques, such as T1 and T2 mapping, offers promising avenues for reducing reliance on contrast agents without compromising diagnostic quality.

### 5.6 Standardization and Technical Variability

Standardization remains an important challenge in the clinical implementation of CMR. Variability in acquisition techniques, reconstruction methods, and post-processing approaches can affect image quality, quantitative measurements, and diagnostic consistency across institutions. Advanced techniques like motion mitigation strategies and non-contrast mapping require specialized reconstruction algorithms that may not be readily available in all clinical settings and contribute to the disparities in accessibility of the imaging modality [27,28,85]. In addition, the effective use of contrast agents depends on standardized patient screening, protocol selection, and individualized risk-benefit assessment with respect to renal function and gadolinium safety [34,93]. These factors highlight the need for continued development of standardized imaging protocols and uniformity of CMR practices in order to ensure consistent diagnostic performance and reliable interpretation across various clinical environments.

## 6. Emerging Trends in Cardiac MRI

With its non-invasive methodology and extensive diagnostic capabilities, CMR has started to play an increasingly important role in modern cardiovascular medicine. Ongoing advancements in imaging techniques, tissue characterization, and integration with emerging technologies continue to enhance its clinical utility and scope. With the emerging trends of precision medicine and hybrid imaging modalities, CMR has the potential to solidify its place as an important imaging modality within the field of medicine.

### 6.1 CMR and Precision Medicine

CMR is undergoing rapid transformation as emerging trends align it more closely with the principles of precision medicine. Precision medicine is the tailoring of medical treatment to the individual characteristics of each patient, including their genetics, environment, and lifestyle [99,100]. Advanced noninvasive imaging modalities, particularly CMR, are emerging as central tools in precision cardiovascular medicine. Techniques such as T1 and T2 mapping, LGE, and extracellular volume quantification generate quantitative biomarkers that, in combination with other data, may enable highly individualized targeted diagnostic and therapeutic approaches [99,101]. Because of its ability to non-invasively characterize myocardial tissue, quantify function, and evaluate perfusion, CMR serves as a powerful tool for informing patient-specific clinical decisions [99,102]. As precision medicine becomes more central to healthcare, CMR’s role in tailoring interventions and forecasting outcomes is expected to expand significantly.

### 6.2 Integration of CMR With Other Imaging Modalities

The integration of CMR with other imaging modalities such as PET, CT, and echocardiography is enhancing diagnostic accuracy and providing a more comprehensive understanding of complex cardiovascular pathologies.

Hybrid imaging techniques, particularly PET/MRI, offer the ability to simultaneously assess structural, functional, and molecular characteristics of the myocardium. This integration combines the high spatial resolution and soft tissue contrast of CMR with the metabolic and molecular insights provided by PET [103]. This offers unique advantages in the evaluation of various cardiovascular diseases, including inflammatory cardiomyopathy, sarcoidosis, coronary artery disease, and ischemic heart disease [103,104,105]. The European Society of Cardiovascular Radiology and the European Association of Nuclear Medicine recognize PET/MRI as a highly promising modality for improving cardiac imaging through greater diagnostic accuracy and enhanced monitoring of treatment responses in cardiovascular disease; however, they emphasize the need for more comprehensive and robust clinical research to fully establish its clinical utility [104]. Similarly, hybrid approaches involving high-resolution CMR and echocardiography allow for more precise anatomical correlation, especially in complex congenital heart disease and valvular defects [106]. Along with the advances in hybrid imaging, there is also increasing interest in CMR not only being a diagnostic tool but also a real-time guide for therapeutic cardiac interventions.

Along with hybrid imaging techniques, the potential of CMR to guide complex cardiac procedures is drawing growing clinical and research interest. As cardiac interventions target increasingly complex conditions, the need for more advanced imaging guidance has become evident. The potential of CMR to be an alternative to conventional X-ray fluoroscopy for guiding these complex cardiac procedures is becoming increasingly more evident [107]. Real-time CMR guidance offers unique advantages, including superior soft tissue contrast, the absence of ionizing radiation, and the ability to visualize both anatomy and function simultaneously during interventions [107].

These integrative approaches exemplify the expanding role of CMR in cardiovascular imaging, creating a space for more personalized and accurate clinical decision-making.

## 7. Conclusion

### 7.1 Summary of Advancements

CMR, as a standard of cardiovascular imaging, continues to improve in various ways. This review has served to address numerous key advancements in CMR. Notable improvements in CMR technology include high-resolution imaging sequences, innovations in T1 and T2 mapping techniques, and 4D flow MRI integration to assess complex hemodynamics at a physiological state. These advances have improved the spatial and temporal resolution of CMR, reducing breath-holding necessity and characterizing vascular and metabolic abnormalities. Further advances in non-gadolinium-based contrast agents and machine learning integration provide safer and more specific tissue analysis and increase read times and standardize reporting measures. Together, these developments expand the clinical utility of CMR, which has enabled more precise diagnosis, risk assessment, and patient management.

### 7.2 Future Research Directions

Despite substantial advances, CMR continues to face challenges related to patient tolerability, cost, access disparities, and the specialized expertise required for full clinical implementation. Addressing these limitations will be essential for full realization of the potential of CMR in improving patient care and expanding its role across diverse healthcare environments.

In response to these challenges, ongoing technical innovation has increasingly focused on improving safety, efficiency, and accessibility while preserving the diagnostic strengths of CMR. Building on advances in parametric mapping and tissue characterization, future research should further refine and validate non-contrast techniques, particularly T1 and T2 mapping, to reduce reliance on gadolinium-based contrast agents while preserving diagnostic accuracy and sensitivity [102]. Native T1 and T2 mapping enable quantitative assessment of intrinsic myocardial relaxation properties, which allows clinicians to detect and characterize diffuse fibrosis, edema, and inflammation without exogenous contrast [102]. These methods are increasingly being incorporated into clinical practice and show promise for identifying early disease processes like subtle tissue changes in myocarditis and other cardiomyopathies. These developments will help mitigate risks associated with gadolinium exposure in patients and concerns regarding gadolinium deposition while supporting safer imaging in patients with renal impairment. As these techniques evolve, their impact will increasingly rely on integration with machine learning and artificial intelligence for automated analysis and enhanced disease characterization.

As CMR techniques generate increasingly large and complex data sets, the ability to efficiently process and interpret this information has become a critical focus of future development. Artificial intelligence and machine learning techniques have the potential to transform CMR workflows by automating segmentation and interpretation, accelerating image reconstruction, improving image quality, and enabling integration with clinical and demographic data for prognostic models and radiomics feature extraction [42,108,109]. Deep learning-based approaches can substantially reduce post-processing time while providing personalized patient care [108]. These advances could mitigate patient-related challenges and workforce constraints, which will help facilitate a broader clinical adoption.

While advances in automation and data analysis address workflow and workforce limitations, parallel efforts are needed to overcome financial and infrastructural barriers for CMR adoption. The development and validation of low-field MRI systems may improve affordability and accessibility by lowering the installation and operational costs while also maintaining clinically usable image quality. Overall, this will potentially make CMR more feasible in community and resource-limited settings, allowing for the expansion of the reach of cardiovascular imaging. As telemedicine continues to increase in the medical sphere, this could allow for CMR expertise to be available in regions without local specialists, helping to bridge the gap between geographic access disparities.

Beyond improving access, expanding the clinical scope of CMR through integration with complementary imaging modalities and procedural guidance represents another important point of growth. Integration with other imaging modalities such as PET and CT holds promise for more comprehensive assessment of cardiac structure, function, metabolism, and perfusion when combined with CMR’s strength in soft tissue characterization and flow quantification [110]. Similarly, the increasing interest in interventional MRI could help aim to reduce reliance on ionizing radiation. This could allow for CMR guidance in procedures that currently are dependent on fluoroscopy.

These technological and clinical integrations further help position CMR as an emerging modality for data-driven cardiovascular care. The trend toward multi-omics integration, which consists of combining radiomic features from CMR with genomic and clinical data, may further enhance disease phenotyping, risk prediction, and personalized management strategies. Early work that links radiomics and clinical prediction models suggests that this integration could substantially improve the classification of disease states and prediction of outcomes, although a larger multicenter validation is still needed [108]. This integration would further cement CMR’s role in precision cardiovascular medicine.

### 7.3 Impact on Patient Outcomes

CMR improvements on patient and clinical outcomes are substantial. Advances in CMR help obtain a more comprehensive understanding of cardiac pathology and facilitate individualized patient care, as CMR helps improve modern cardiovascular medicine, ultimately improving diagnostic precision and patient outcomes.

## References

[1] Global Burden of Cardiovascular Diseases and Risks 2023 Collaborators (2025). Global, Regional, and National Burden of Cardiovascular Diseases and Risk Factors in 204 Countries and Territories, 1990-2023. Journal of the American College of Cardiology.

[2] Roth GA, Mensah GA, Johnson CO, Addolorato G, Ammirati E, Baddour LM (2020). Global Burden of Cardiovascular Diseases and Risk Factors, 1990-2019: Update From the GBD 2019 Study. Journal of the American College of Cardiology.

[3] Joynt Maddox KE, Elkind MS, Aparicio HJ, Commodore-Mensah Y, de Ferranti SD, Dowd WN (2024). Forecasting the Burden of Cardiovascular Disease and Stroke in the United States Through 2050—Prevalence of Risk Factors and Disease: A Presidential Advisory From the American Heart Association. Circulation.

[4] Chong B, Jayabaskaran J, Jauhari SM, Chan SP, Goh R, Kueh MTW (2025). Global burden of cardiovascular diseases: projections from 2025 to 2050. European Journal of Preventive Cardiology.

[5] Laslett LJ, Alagona P, Clark BA, Drozda JP, Saldivar F, Wilson SR (2012). The worldwide environment of cardiovascular disease: prevalence, diagnosis, therapy, and policy issues: a report from the American College of Cardiology. Journal of the American College of Cardiology.

[6] Hundley WG (2024). Fifty Years of Cardiovascular Magnetic Resonance: Continuing Evolution Toward the "One-Stop Shop" for Cardiovascular Diagnosis. Circulation.

[7] Rajiah PS, François CJ, Leiner T (2023). Cardiac MRI: State of the Art. Radiology.

[8] Sanghvi MM, Lima JAC, Bluemke DA, Petersen SE (2024). A history of cardiovascular magnetic resonance imaging in clinical practice and population science. Frontiers in Cardiovascular Medicine.

[9] Bhatt AB, Foster E, Kuehl K, Alpert J, Brabeck S, Crumb S (2015). Congenital heart disease in the older adult: a scientific statement from the American Heart Association. Circulation.

[10] Vaz A, Serra VC, de Santana Ramos DO (2025). Beyond volumes and ejection fraction: practical insights into cine-CMR interpretation and applications. The International Journal of Cardiovascular Imaging.

[11] Wang X, Uecker M, Feng L (2021). Fast Real-Time Cardiac MRI: a Review of Current Techniques and Future Directions. Investigative Magnetic Resonance Imaging.

[12] Wang Z, Wang F, Qin C, Lyu J, Ouyang C, Wang S (2025). CMRxRecon2024: A Multimodality, Multiview k-Space Dataset Boosting Universal Machine Learning for Accelerated Cardiac MRI. Radiology. Artificial Intelligence.

[13] Haaf P, Garg P, Messroghli DR, Broadbent DA, Greenwood JP, Plein S (2016). Cardiac T1 Mapping and Extracellular Volume (ECV) in clinical practice: a comprehensive review. Journal of Cardiovascular Magnetic Resonance.

[14] Giri S, Chung YC, Merchant A, Mihai G, Rajagopalan S, Raman SV (2009). T2 quantification for improved detection of myocardial edema. Journal of Cardiovascular Magnetic Resonance.

[15] Ricco A, Slade A, Canada JM, Grizzard J, Dana F, Rezai Gharai L (2020). Cardiac MRI utilizing late gadolinium enhancement (LGE) and T1 mapping in the detection of radiation induced heart disease. Cardio-Oncology.

[16] Messroghli DR, Moon JC, Ferreira VM, Grosse-Wortmann L, He T, Kellman P (2017). Clinical recommendations for cardiovascular magnetic resonance mapping of T1, T2, T2* and extracellular volume: A consensus statement by the Society for Cardiovascular Magnetic Resonance (SCMR) endorsed by the European Association for Cardiovascular Imaging (EACVI). Journal of Cardiovascular Magnetic Resonance.

[17] Schelbert EB, Messroghli DR (2016). State of the Art: Clinical Applications of Cardiac T1 Mapping. Radiology.

[18] Warnica W, Al-Arnawoot A, Stanimirovic A, Thavendiranathan P, Wald RM, Pakkal M (2022). Clinical Impact of Cardiac MRI T1 and T2 Parametric Mapping in Patients with Suspected Cardiomyopathy. Radiology.

[19] Gao Y, Wang HP, Liu MX, Gu H, Yuan XS, Biekan J (2023). Early detection of myocardial fibrosis in cardiomyopathy in the absence of late enhancement: role of T1 mapping and extracellular volume analysis. European Radiology.

[20] Ferreira VM, Schulz-Menger J, Holmvang G, Kramer CM, Carbone I, Sechtem U (2018). Cardiovascular Magnetic Resonance in Nonischemic Myocardial Inflammation: Expert Recommendations. Journal of the American College of Cardiology.

[21] Giusca S, Steen H, Montenbruck M, Patel AR, Pieske B, Erley J (2021). Multi-parametric assessment of left ventricular hypertrophy using late gadolinium enhancement, T1 mapping and strain-encoded cardiovascular magnetic resonance. Journal of Cardiovascular Magnetic Resonance.

[22] Rajiah PS, Kalisz K, Broncano J, Goerne H, Collins JD, François CJ (2022). Myocardial Strain Evaluation with Cardiovascular MRI: Physics, Principles, and Clinical Applications. Radiographics.

[23] Smiseth OA, Rider O, Cvijic M, Valkovič L, Remme EW, Voigt JU (2025). Myocardial Strain Imaging: Theory, Current Practice, and the Future. JACC. Cardiovascular Imaging.

[24] von Knobelsdorff-Brenkenhoff F, Schunke T, Reiter S, Scheck R, Höfling B, Pilz G (2020). Influence of contrast agent and spatial resolution on myocardial strain results using feature tracking MRI. European Radiology.

[25] Soulat G, McCarthy P, Markl M (2020). 4D Flow with MRI. Annual Review of Biomedical Engineering.

[26] Peper ES, van Ooij P, Jung B, Huber A, Gräni C, Bastiaansen JAM (2022). Advances in machine learning applications for cardiovascular 4D flow MRI. Frontiers in Cardiovascular Medicine.

[27] Solomon O, Patriat R, Braun H, Palnitkar TE, Moeller S, Auerbach EJ (2023). Motion robust magnetic resonance imaging via efficient Fourier aggregation. Medical Image Analysis.

[28] Nayak KS, Lim Y, Campbell-Washburn AE, Steeden J (2022). Real-Time Magnetic Resonance Imaging. Journal of Magnetic Resonance Imaging.

[29] Ismail TF, Strugnell W, Coletti C, Božić-Iven M, Weingärtner S, Hammernik K (2022). Cardiac MR: From Theory to Practice. Frontiers in Cardiovascular Medicine.

[30] Cheng HLM (2022). Emerging MRI techniques for molecular and functional phenotyping of the diseased heart. Frontiers in Cardiovascular Medicine.

[31] Tsampasian V, Swift AJ, Assadi H, Chowdhary A, Swoboda P, Sammut E (2021). Myocardial inflammation and energetics by cardiac MRI: a review of emerging techniques. BMC Medical Imaging.

[32] Backhaus SJ, Metschies G, Billing M, Schmidt-Rimpler J, Kowallick JT, Gertz RJ (2021). Defining the optimal temporal and spatial resolution for cardiovascular magnetic resonance imaging feature tracking. Journal of Cardiovascular Magnetic Resonance.

[33] Bustin A, Fuin N, Botnar RM, Prieto C (2020). From Compressed-Sensing to Artificial Intelligence-Based Cardiac MRI Reconstruction. Frontiers in Cardiovascular Medicine.

[34] American College of Radiology (2025). ACR Manual on Contrast Media. https://www.acr.org/Clinical-Resources/Clinical-Tools-and-Reference/Contrast-Manual.

[35] Caspani S, Magalhães R, Araújo JP, Sousa CT (2020). Magnetic Nanomaterials as Contrast Agents for MRI. Materials.

[36] Fernández-Barahona I, Muñoz-Hernando M, Ruiz-Cabello J, Herranz F, Pellico J (2020). Iron Oxide Nanoparticles: An Alternative for Positive Contrast in Magnetic Resonance Imaging. Inorganics.

[37] Al-Shimmari HA, Ab Hamid S, Suppiah S, Kadhim DA, Zyoud TY, Abdullah WH (2025). Beyond Gadolinium: A Comparative Review of Iron Oxide Nanoparticles as Emerging MRI Contrast Agents for Personalized Medicine. South Eastern European Journal of Public Health.

[38] Bendszus M, Laghi A, Munuera J, Tanenbaum LN, Taouli B, Thoeny HC (2024). MRI Gadolinium-Based Contrast Media: Meeting Radiological, Clinical, and Environmental Needs. Journal of Magnetic Resonance Imaging.

[39] Poggiarelli L, Bernetti C, Pugliese L, Greco F, Beomonte Zobel B, Mallio CA (2025). Manganese-Based Contrast Agents as Alternatives to Gadolinium: A Comprehensive Review. Clinics and Practice.

[40] Amano Y, Suzuki Y, Tang X, Ando C (2025). Identifying etiologies of heart failure using non-contrast cardiac magnetic resonance imaging: cine imaging, T1 and T2 mapping, and texture analysis for T1 mapping. Frontiers in Cardiovascular Medicine.

[41] Argentiero A, Muscogiuri G, Rabbat MG, Martini C, Soldato N, Basile P (2022). The Applications of Artificial Intelligence in Cardiovascular Magnetic Resonance-A Comprehensive Review. Journal of Clinical Medicine.

[42] Morales MA, Manning WJ, Nezafat R (2024). Present and Future Innovations in AI and Cardiac MRI. Radiology.

[43] Alabed S, Alandejani F, Dwivedi K, Karunasaagarar K, Sharkey M, Garg P (2022). Validation of Artificial Intelligence Cardiac MRI Measurements: Relationship to Heart Catheterization and Mortality Prediction. Radiology.

[44] Le Y, Zhao C, An J, Zhou J, Deng D, He Y (2024). Progress in the Clinical Application of Artificial Intelligence for Left Ventricle Analysis in Cardiac Magnetic Resonance. Reviews in Cardiovascular Medicine.

[45] Hadamitzky M, Bressem KK, Dürner C, Hendrich E, Adolf R, Stambollxhiu E (2025). AI-based myocardial segmentation and cardiac disease classification using multi-sequence cardiac MRI. Journal of Cardiovascular Magnetic Resonance.

[46] Wang YRJ, Yang K, Wen Y, Wang P, Hu Y, Lai Y (2024). Screening and diagnosis of cardiovascular disease using artificial intelligence-enabled cardiac magnetic resonance imaging. Nature Medicine.

[47] Tolu-Akinnawo OZ, Ezekwueme F, Omolayo O, Batheja S, Awoyemi T (2025). Advancements in Artificial Intelligence in Noninvasive Cardiac Imaging: A Comprehensive Review. Clinical Cardiology.

[48] Alsharqi M, Edelman ER (2025). Artificial Intelligence in Cardiovascular Imaging and Interventional Cardiology: Emerging Trends and Clinical Implications. Journal of the Society for Cardiovascular Angiography & Interventions.

[49] Lewin S, Chetty R, Ihdayhid AR, Dwivedi G (2024). Ethical Challenges and Opportunities in Applying Artificial Intelligence to Cardiovascular Medicine. The Canadian Journal of Cardiology.

[50] Mastrodicasa D, van Assen M, Huisman M, Leiner T, Williamson EE, Nicol ED (2025). Use of AI in Cardiac CT and MRI: A Scientific Statement from the ESCR, EuSoMII, NASCI, SCCT, SCMR, SIIM, and RSNA. Radiology.

[51] Mentias A, Raeisi-Giglou P, Smedira NG, Feng K, Sato K, Wazni O (2018). Late Gadolinium Enhancement in Patients With Hypertrophic Cardiomyopathy and Preserved Systolic Function. Journal of the American College of Cardiology.

[52] Aquaro GD, Todiere G, Barison A, Grigoratos C, Parisella ML, Adami M (2024). Prognostic Role of the Progression of Late Gadolinium Enhancement in Hypertrophic Cardiomyopathy. The American Journal of Cardiology.

[53] Halliday BP, Gulati A, Ali A, Guha K, Newsome S, Arzanauskaite M (2017). Association Between Midwall Late Gadolinium Enhancement and Sudden Cardiac Death in Patients With Dilated Cardiomyopathy and Mild and Moderate Left Ventricular Systolic Dysfunction. Circulation.

[54] Halliday BP, Baksi AJ, Gulati A, Ali A, Newsome S, Izgi C (2019). Outcome in Dilated Cardiomyopathy Related to the Extent, Location, and Pattern of Late Gadolinium Enhancement. JACC. Cardiovascular Imaging.

[55] Fontana M, Pica S, Reant P, Abdel-Gadir A, Treibel TA, Banypersad SM (2015). Prognostic Value of Late Gadolinium Enhancement Cardiovascular Magnetic Resonance in Cardiac Amyloidosis. Circulation.

[56] Banypersad SM, Fontana M, Maestrini V, Sado DM, Captur G, Petrie A (2015). T1 mapping and survival in systemic light-chain amyloidosis. European Heart Journal.

[57] O'Brien AT, Gil KE, Varghese J, Simonetti OP, Zareba KM (2022). T2 mapping in myocardial disease: a comprehensive review. Journal of Cardiovascular Magnetic Resonance.

[58] Souto ALM, Souto RM, Teixeira ICR, Nacif MS (2017). Myocardial Viability on Cardiac Magnetic Resonance. Arquivos Brasileiros De Cardiologia.

[59] Scatteia A, Dellegrottaglie S (2023). Cardiac magnetic resonance in ischemic cardiomyopathy: present role and future directions. European Heart Journal Supplements: Journal of the European Society of Cardiology.

[60] Li M, Zhou T, Yang LF, Peng ZH, Ding J, Sun G (2014). Diagnostic accuracy of myocardial magnetic resonance perfusion to diagnose ischemic stenosis with fractional flow reserve as reference: systematic review and meta-analysis. JACC. Cardiovascular Imaging.

[61] Yang K, Yu SQ, Lu MJ, Zhao SH (2019). Comparison of diagnostic accuracy of stress myocardial perfusion imaging for detecting hemodynamically significant coronary artery disease between cardiac magnetic resonance and nuclear medical imaging: A meta-analysis. International Journal of Cardiology.

[62] Zhao X, Zeng D, He L, Sun W (2022). Clinical and imaging characteristics of cardiac magnetic resonance presenting with myocardial infarction with non-obstructive coronary arteries in China. Journal of Cardiothoracic Surgery.

[63] Tornvall P, Beltrame JF, Nickander J, Sörensson P, Reynolds HR, Agewall S (2024). How to Use Cardiac Magnetic Resonance Imaging in Myocardial Infarction With Nonobstructive Coronary Arteries. Circulation. Cardiovascular Imaging.

[64] Dastidar AG, Singhal P, Rodrigues JC, Ahmed N, Palazzuoli A, Townsend M (2015). Improved diagnostic role of CMR in acute coronary syndromes and unobstructed coronary arteries: the importance of time-to-CMR. Journal of Cardiovascular Magnetic Resonance.

[65] Driessen MMP, Breur JMPJ, Budde RPJ, van Oorschot JWM, van Kimmenade RRJ, Sieswerda GT (2015). Advances in cardiac magnetic resonance imaging of congenital heart disease. Pediatric Radiology.

[66] Saraya S, Woodard P, Bhalla S, Gutierrez F, Saraya M, Soliman HH (2020). Cardiac MRI in evaluation of post-operative congenital heart disease and complications. Egyptian Journal of Radiology and Nuclear Medicine.

[67] Kotecha T, Knight DS, Razvi Y, Kumar K, Vimalesvaran K, Thornton G (2021). Patterns of myocardial injury in recovered troponin-positive COVID-19 patients assessed by cardiovascular magnetic resonance. European Heart Journal.

[68] Aquaro GD, De Luca A, Cappelletto C, Raimondi F, Bianco F, Botto N (2020). Prognostic Value of Magnetic Resonance Phenotype in Patients With Arrhythmogenic Right Ventricular Cardiomyopathy. Journal of the American College of Cardiology.

[69] Rastegar N, Te Riele ASJM, James CA, Bhonsale A, Murray B, Tichnell C (2016). Fibrofatty Changes: Incidence at Cardiac MR Imaging in Patients with Arrhythmogenic Right Ventricular Dysplasia/Cardiomyopathy. Radiology.

[70] Soto-Iglesias D, Penela D, Jáuregui B, Acosta J, Fernández-Armenta J, Linhart M (2020). Cardiac Magnetic Resonance-Guided Ventricular Tachycardia Substrate Ablation. JACC. Clinical Electrophysiology.

[71] Mont L, Roca-Luque I, Althoff TF (2022). Ablation Lesion Assessment with MRI. Arrhythmia & Electrophysiology Review.

[72] Nakamori S, Dohi K, Ishida M, Goto Y, Imanaka-Yoshida K, Omori T (2018). Native T1 Mapping and Extracellular Volume Mapping for the Assessment of Diffuse Myocardial Fibrosis in Dilated Cardiomyopathy. JACC. Cardiovascular Imaging.

[73] Puntmann VO, Carr-White G, Jabbour A, Yu CY, Gebker R, Kelle S (2016). T1-Mapping and Outcome in Nonischemic Cardiomyopathy: All-Cause Mortality and Heart Failure. JACC. Cardiovascular Imaging.

[74] Raisi-Estabragh Z, McCracken C, Hann E, Condurache DG, Harvey NC, Munroe PB (2023). Incident Clinical and Mortality Associations of Myocardial Native T1 in the UK Biobank. JACC. Cardiovascular Imaging.

[75] Spieker M, Haberkorn S, Gastl M, Behm P, Katsianos S, Horn P (2017). Abnormal T2 mapping cardiovascular magnetic resonance correlates with adverse clinical outcome in patients with suspected acute myocarditis. Journal of Cardiovascular Magnetic Resonance.

[76] Zavar R, Hendimarjan M, Behjati M, Yazdani D (2022). T2-weighted cardiovascular magnetic resonance and echocardiographic arterial elasticity criteria for monitoring cardiac siderosis in patients with beta-thalassemia major. Journal of Research in Medical Sciences: the Official Journal of Isfahan University of Medical Sciences.

[77] Weng Z, Yao J, Chan RH, He J, Yang X, Zhou Y (2016). Prognostic Value of LGE-CMR in HCM: A Meta-Analysis. JACC. Cardiovascular Imaging.

[78] Sammut EC, Villa ADM, Di Giovine G, Dancy L, Bosio F, Gibbs T (2018). Prognostic Value of Quantitative Stress Perfusion Cardiac Magnetic Resonance. JACC. Cardiovascular Imaging.

[79] Cai S, Haghbayan H, Chan KKW, Deva DP, Jimenez-Juan L, Connelly KA (2024). Tissue mapping by cardiac magnetic resonance imaging for the prognostication of cardiac amyloidosis: A systematic review and meta-analysis. International Journal of Cardiology.

[80] Sammet S (2016). Magnetic resonance safety. Abdominal Radiology (New York).

[81] Madl J, Janka R, Bay S, Rohleder N (2022). MRI as a Stressor: The Psychological and Physiological Response of Patients to MRI, Influencing Factors, and Consequences. Journal of the American College of Radiology: JACR.

[82] Saloner D, Liu J, Haraldsson H (2015). MR physics in practice: how to optimize acquisition quality and time for cardiac MR imaging. Magnetic Resonance Imaging Clinics of North America.

[83] Sheagren CD, Cao T, Patel JH, Chen Z, Lee HL, Wang N (2023). Motion-compensated T_1_ mapping in cardiovascular magnetic resonance imaging: a technical review. Frontiers in Cardiovascular Medicine.

[84] Rajiah PS, Sundaram B, Ng MY, Ranganath P, Araoz PA, Bolen MA (2025). Artifacts at Cardiac MRI: Imaging Appearances and Solutions. Radiographics.

[85] Li JM, Ho DR, Husain N, Biederman RW, Finn JP, Fuisz AR (2024). Regional variability of cardiovascular magnetic resonance access and utilization in the United States. Journal of Cardiovascular Magnetic Resonance.

[86] Parwani P, Chen T, Allen B, Kallianos K, Ng MY, Kozor R (2023). Challenges and opportunities for early career medical professionals in cardiovascular magnetic resonance (CMR) imaging: a white paper from the Society for Cardiovascular Magnetic Resonance. Journal of Cardiovascular Magnetic Resonance.

[87] Qin C, Murali S, Lee E, Supramaniam V, Hausenloy DJ, Obungoloch J (2022). Sustainable low-field cardiovascular magnetic resonance in changing healthcare systems. European Heart Journal. Cardiovascular Imaging.

[88] Geethanath S, Vaughan JT (2019). Accessible magnetic resonance imaging: A review. Journal of Magnetic Resonance Imaging: JMRI.

[89] Arrighi JA, Kilic S, Haines PG (2018). Perspectives on Current Training Guidelines for Cardiac Imaging and Recommendations for the Future. Current Cardiology Reports.

[90] Patel N, Kolakalapudi P, Arora G (2018). Contrast - in cardiac magnetic resonance imaging. Echocardiography.

[91] Paiman EHM, Lamb HJ (2017). When should we use contrast material in cardiac MRI?. Journal of Magnetic Resonance Imaging: JMRI.

[92] Shahid I, Morvan J, Darmon-Kern E, Hebert F, Lancelot E, Bourrinet P (2025). Safety of Gadoterate Meglumine: A Review of 35 Years of Clinical Use and More Than 170 Million Doses. Investigative Radiology.

[93] Palamuthusingam D, Reyaldeen R, Johnson DW, Hawley CM, Pascoe EM, Wahi S (2021). Assessment of cardiac structure and function in kidney failure: understanding echocardiography and magnetic resonance imaging for the nephrologist. International Urology and Nephrology.

[94] Gallo-Bernal S, Patino-Jaramillo N, Calixto CA, Higuera SA, Forero JF, Lara Fernandes J (2022). Nephrogenic Systemic Fibrosis in Patients with Chronic Kidney Disease after the Use of Gadolinium-Based Contrast Agents: A Review for the Cardiovascular Imager. Diagnostics.

[95] Lohrke J, Frenzel T, Endrikat J, Alves FC, Grist TM, Law M (2016). 25 Years of Contrast-Enhanced MRI: Developments, Current Challenges and Future Perspectives. Advances in Therapy.

[96] Gulani V, Calamante F, Shellock FG, Kanal E, Reeder SB, International Society for Magnetic Resonance in Medicine (2017). Gadolinium deposition in the brain: summary of evidence and recommendations. The Lancet. Neurology.

[97] McDonald RJ, McDonald JS, Kallmes DF, Jentoft ME, Murray DL, Thielen KR (2015). Intracranial Gadolinium Deposition after Contrast-enhanced MR Imaging. Radiology.

[98] Murata N, Gonzalez-Cuyar LF, Murata K, Fligner C, Dills R, Hippe D (2016). Macrocyclic and Other Non-Group 1 Gadolinium Contrast Agents Deposit Low Levels of Gadolinium in Brain and Bone Tissue: Preliminary Results From 9 Patients With Normal Renal Function. Investigative Radiology.

[99] Achenbach S, Fuchs F, Goncalves A, Kaiser-Albers C, Ali ZA, Bengel FM (2022). Non-invasive imaging as the cornerstone of cardiovascular precision medicine. European Heart Journal. Cardiovascular Imaging.

[100] Tingen HSA, van Praagh GD, Nienhuis PH, Tubben A, van Rijsewijk ND, Ten Hove D (2023). The clinical value of quantitative cardiovascular molecular imaging: a step towards precision medicine. The British Journal of Radiology.

[101] Ascione R, De Giorgi M, Dell’Aversana S, Di Costanzo G, Nappi C, Imbriaco M (2023). The Additional Value of T1 Mapping in Cardiac Disease: State of the Art. Current Cardiovascular Imaging Reports.

[102] Simkowski J, Eck B, Tang WHW, Nguyen C, Kwon DH (2024). Next-Generation Cardiac Magnetic Resonance Imaging Techniques for Characterization of Myocardial Disease. Current Treatment Options in Cardiovascular Medicine.

[103] Fukushima K, Ito H, Takeishi Y (2023). Comprehensive assessment of molecular function, tissue characterization, and hemodynamic performance by non-invasive hybrid imaging: Potential role of cardiac PETMR. Journal of Cardiology.

[104] Nensa F, Bamberg F, Rischpler C, Menezes L, Poeppel TD, la Fougère C (2018). Hybrid cardiac imaging using PET/MRI: a joint position statement by the European Society of Cardiovascular Radiology (ESCR) and the European Association of Nuclear Medicine (EANM). European Radiology.

[105] Aitken M, Chan MV, Urzua Fresno C, Farrell A, Islam N, McInnes MDF (2022). Diagnostic Accuracy of Cardiac MRI versus FDG PET for Cardiac Sarcoidosis: A Systematic Review and Meta-Analysis. Radiology.

[106] Gomez A, Gomez G, Simpson J, Valverde I (2020). 3D hybrid printed models in complex congenital heart disease: 3D echocardiography and cardiovascular magnetic resonance imaging fusion. European Heart Journal.

[107] Campbell-Washburn AE, Tavallaei MA, Pop M, Grant EK, Chubb H, Rhode K (2017). Real-time MRI guidance of cardiac interventions. Journal of Magnetic Resonance Imaging: JMRI.

[108] Jiménez-Jara C, Salas R, Díaz-Navarro R, Chabert S, Andia ME, Vega J (2025). AI Applied to Cardiac Magnetic Resonance for Precision Medicine in Coronary Artery Disease: A Systematic Review. Journal of Cardiovascular Development and Disease.

[109] Cicek V, Bagci U (2024). AI-powered contrast-free cardiovascular magnetic resonance imaging for myocardial infarction. Frontiers in Cardiovascular Medicine.

[110] Zhou Y, Lin H, Huang C, Xu Y, Li Y, Xia C (2025). An innovative hybrid imaging protocol for whole-body ^18^F-FDG-PET/MRI in cardio-oncology: a proof-of-concept study. Cardio-Oncology.

